# Phenotypic CD8 T cell profiling in chronic hepatitis B to predict HBV-specific CD8 T cell susceptibility to functional restoration in vitro

**DOI:** 10.1136/gutjnl-2022-327202

**Published:** 2023-01-30

**Authors:** Marzia Rossi, Andrea Vecchi, Camilla Tiezzi, Valeria Barili, Paola Fisicaro, Amalia Penna, Ilaria Montali, Stephane Daffis, Simon P Fletcher, Anuj Gaggar, Jonathan Medley, Michael Graupe, Latesh Lad, Alessandro Loglio, Roberta Soffredini, Marta Borghi, Teresa Pollicino, Cristina Musolino, Arianna Alfieri, Federica Brillo, Diletta Laccabue, Marco Massari, Chiara Boarini, Gianluca Abbati, Giuseppe Pedrazzi, Gabriele Missale, Pietro Lampertico, Carlo Ferrari, Carolina Boni

**Affiliations:** 1 Department of Medicine and Surgery, University of Parma, Parma, Italy; 2 Unit of Infectious Diseases and Hepatology, Azienda Ospedaliero-Universitaria di Parma, Parma, Italy; 3 Gilead Sciences Inc, Foster City, California, USA; 4 Division of Gastroenterology and Hepatology, Foundation IRCCS Ca’ Granda Ospedale Maggiore Policlinico, Milano, Italy; 5 Department of Human Pathology, University Hospital of Messina, Messina, Italy; 6 Unit of Infectious Diseases, IRCCS, Reggio Emilia, Italy; 7 Division of Internal Medicine 2 and Center for Hemochromatosis, University of Modena and Reggio Emilia, Modena, Italy; 8 Department of Neuroscience - Biophysics and Medical Physics Unit, University of Parma, Parma, Italy; 9 Department of Pathophysiology and Transplantation, CRC “A. M. and A. Migliavacca” Center for Liver Disease, Milano, Italy

**Keywords:** chronic viral hepatitis, immune response, cellular immunity, T lymphocytes

## Abstract

**Objective:**

Exhausted hepatitis B virus (HBV)-specific CD8 T cells in chronic HBV infection are broadly heterogeneous. Characterisation of their functional impairment may allow to distinguish patients with different capacity to control infection and reconstitute antiviral function.

**Design:**

HBV dextramer+CD8 T cells were analysed ex vivo for coexpression of checkpoint/differentiation markers, transcription factors and cytokines in 35 patients with HLA-A2+chronic hepatitis B (CHB) and in 29 control HBsAg negative CHB patients who seroconverted after NUC treatment or spontaneously. Cytokine production was also evaluated in HBV peptide-stimulated T cell cultures, in the presence or absence of antioxidant, polyphenolic, PD-1/PD-L1 inhibitor and TLR-8 agonist compounds and the effect on HBV-specific responses was further validated on additional 24 HLA-A2 negative CHB patients.

**Results:**

Severely exhausted HBV-specific CD8 T cell subsets with high expression of inhibitory receptors, such as PD-1, TOX and CD39, were detected only in a subgroup of chronic viraemic patients. Conversely, a large predominance of functionally more efficient HBV-specific CD8 T cell subsets with lower expression of coinhibitory molecules and better response to in vitro immune modulation, typically detected after resolution of infection, was also observed in a proportion of chronic viraemic HBV patients. Importantly, the same subset of patients who responded more efficiently to in vitro immune modulation identified by HBV-specific CD8 T cell analysis were also identified by staining total CD8 T cells with PD-1, TOX, CD127 and Bcl-2.

**Conclusions:**

The possibility to distinguish patient cohorts with different capacity to respond to immune modulatory compounds in vitro by a simple analysis of the phenotypic CD8 T cell exhaustion profile deserves evaluation of its clinical applicability.

**WHAT IS ALREADY KNOWN ON THIS TOPIC**

- Hepatitis B virus (HBV)-specific CD8 T cells play a crucial role in antiviral protection and failure to control HBV is associated with a severe impairment of virus-specific CD8 T cell responses.
- Exhausted HBV-specific CD8 T cells are heterogeneous in chronic HBV infection.

**WHAT THIS STUDY ADDS**

- Simultaneous staining of HBV-specific CD8 T cells with exhaustion and differentiation/memory markers allows to define a phenotypic score for the classification of chronic hepatitis B patients according to the degree of HBV-specific CD8 T cell exhaustion.
- Calculation of this score in individual patients can distinguish different cohorts of patients with distinct probability of response to immune modulatory compounds in vitro.
- Costaining with PD-1, TOX, CD127 and Bcl-2 gives similar results when applied to the total CD8 T cell population providing a simplified approach to predict individual HBV-specific CD8 T cell functionality without requiring direct analysis of virus-specific CD8 T cells.

**HOW THIS STUDY MIGHT AFFECT RESEARCH, PRACTICE OR POLICY**

- In vitro development of an exhaustion score based on total instead of HBV-specific CD8 T cell phenotyping represents a promising experimental basis in the perspective of novel T cell-based diagnostic predictors.

## Introduction

In hepatitis B, chronic evolution of infection is associated with impaired hepatitis B virus (HBV)-specific CD8 T cell responses which are believed to play a key role in the pathogenesis of virus control and persistence. For this reason, a possible therapeutic strategy for chronic HBV infection is to reconstitute an efficent HBV-specific CD8 T cell function.^1 2^

Available data indicate that exhausted virus-specific CD8 T cells are heterogeneous in their antiviral activity and different studies in mouse chronic lymphocytic choriomeningitis virus infection and in human chronic HCV infection indicate that simultaneous detection of different transcription factors, coinhibitory receptors and differentiation molecules can allow to distinguish T cell subsets with different degree of functional impairement and different propensity to reconstitute their antiviral activity.^3–11^

Recently, novel information about the heterogeneity of exhausted virus-specific CD8 T cells was also reported in chronic HBV infection.^12 13^ Even though the HBV-specific T cell population appears globally dysfunctional, different levels of impairment have been reported for T cell subsets targeting different HBV antigen specificities with higher expression of exhaustion markers and lower expansion capacity of polymerase-specific compared with core-specific CD8 T cells.^14–17^

Based on this background, the aim of our study was to characterise in better detail the functional heterogeneity of HBV-specific and total CD8 T cells in chronic hepatitis B (CHB) patients, to assess whether this can affect individual responses to immune modulatory strategies and to identify CD8 T cell functional and phenotypic parameters to quantify HBV-specific T cell impairment in individual patients in the perspective of novel diagnostic tools based on T cell analysis.

## Materials and methods

### Patient populations

**Figure 1:**
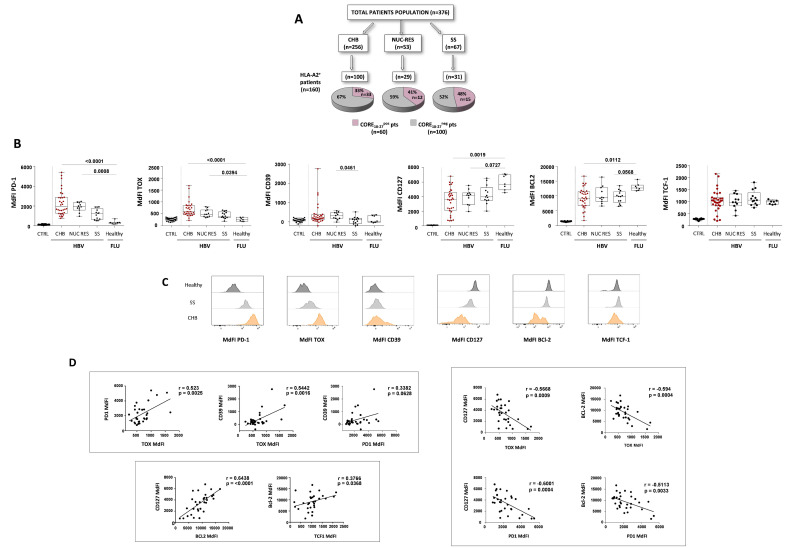
Phenotypical analysis of HBV-specific CD8 T cells. (A) Flow chart of the patient cohorts enrolled in the study. Pie plots indicate core_18-27_-dextramer+ CD8 T cell frequency. (B) PD-1, TOX, CD39, CD127, Bcl-2 and TCF1 MdFI of HBV-core_18-27_ and influenza-specific CD8 T cells in the indicated study groups and in the negative CD8 population calculated for each specific marker within the total CD8+ T cells (negative control). Box-whisker plots show median values and 5th/95th percentiles; each dot represents a single patient. Statistics by the Kruskal-Wallis with Dunn’s correction test. (C) Representative histogram plots for each parameter. (D) Correlation of exhaustion and differentiation/memory markers by core_18-27_-specific CD8 T cells in CHB patients. Statistics by the Spearman’s correlation test. CHB, chronic hepatitis B; HBV, hepatitis B virus; MdFI, median fluorescence intensity; NUC RES, NUC resolved patients; SS, spontaneous seroconversion.

A total of 376 CHB patients were enrolled and screened for HLA-A2 expression. A total of 160 resulted HLA-A2+ and 60 of them showed detectable frequencies of core_18-27_ dextramer+CD8 T cells (figure 1A). Seventy-one HLA-A2+patients were tested also with a polymerase-specific dextramer containing the pol sequence 455–463 and 23 of them came out to be positive. All patients who showed the presence of at least one of the two tested CD8 T cell epitopes (64 patients) represent the final HLA-A2+study population, which comprises the following categories of patients (see also table 1 and online supplemental material):

10.1136/gutjnl-2022-327202.supp1 Supplementary data

- Thirty-five treatment-naïve patients (CHB).
- Sixteen immune subjects spontaneously recovered from chronic HBV infection (spontaneous seroconversion, SS).
- Thirteen NUC resolved patients, as shown by HBsAg clearance following NUC therapy (NUC-RES).

**Table 1:** Demographic and clinical details of different HLA-A2+ patient categories

	Patient ID	Gender	Age (years)	Genotype	Core_18-27_ Sequence FLPSDFFPSV	Therapy	HBsAg (IU/mL)	anti HBs (UI/mL)	HBeAg (IU/ml)	ALT (IU/L)	HBV-DNA (UI/mL)	Detectable CD8+T cell response	
												Core_18-27_	Pol_455-463_
CHB	CHB 1	M	68	D	---**T**-----**A**	Naive	23 549	–	–	52	14 840	+	–
	CHB 2	M	35	D	---**G**------	Naive	1932	–	–	136	237 528	+	–
	CHB 3	M	36	B	---**A**------	Naive	3472	–	–	607	8 132 680	+	+
	CHB 4	F	51	D	----------	Naive	482	–	–	43	66 400	+	–
	CHB 5	F	40	D	---**Q**------	Naive	5398	–	–	168	842 528	+	–
	CHB 6	F	57	A	---**H**------	Naive	4600	–	–	65	229 402	+	–
	CHB 7	M	54	D	----------	Naive	694	–	–	183	9856	+	–
	CHB 8	F	40	D	----------	Naive	608	–	–	93	881 000	+	n.d.
	CHB 9	F	43	D	---**A**--**Y**---	Naive	714	–	–	30	11 453	+	–
	CHB 10	M	34	D	--------**A**-	Naive	4085	–	–	85	3 497 520	+	n.d.
	CHB 11	M	44	D	----------	Naive	8819	–	–	49	20 700	+	+
	CHB 12	M	46	n.d.	n.d.	Naive	+	–	–	133	n.d.	+	+
	CHB 13	M	64	A	---**A**-----**I**	Naive	185	–	–	33	5140	+	–
	CHB 14	F	29	D	---**T**--**Y**---	Naive	5139.73	–	–	21	31 721	+	–
	CHB 15	F	57	D	----------	Naive	19 679	–	–	34	509 000	+	+
	CHB 16	M	34	D	--------**P**-	Naive	2752	–	–	40	86 620	+	n.d.
	CHB 17	F	42	C	----------	Naive	144	–	+	51	6 800 000	+	+
	CHB 18	M	54	D	----------	Naive	4775	–	–	68	153 246	+	–
	CHB 19	M	71	D	---**A**------	Naive	n.d.	–	–	165	260 000	+	n.d.
	CHB 20	M	52	D	------**Y**---	Naive	2662	–	–	40	21 331	+	–
	CHB 21	M	44	D	---**N**-----**A**	Naive	3736	–	–	146	1 400 591	+	–
	CHB 22*	F	66	D	---------**I**	Naive	98	148	–	23	7744	+	–
	CHB 23	F	26	A	----------	Naive	7171.45	–	–	12	3380	+	+
	CHB 24	M	50	D	---**A**--**Y**---	Naive	2940	–	–	35	9733	+	–
	CHB 25	M	45	n.d.	---------**I**	Naive	10 539.72	–	+	123	999 848	+	–
	CHB 26	M	38	D	---**H**-----**I**	Naive	5031.25	–	–	57	110 009	+	–
	CHB 27	M	27	E	---**A**------	Naive	30 915.9	–	–	41	23 337	+	–
	CHB 28	M	45	D	---**A**------	Naive	1764	–	–	16	7760	+	+
	CHB 29	M	71	D	---**A**--**Y**---	Naive	3097	–	–	23	5030	+	+
	CHB 30	M	44	D	n.d.	Naive	0.57	–	–	33	283	+	+
	CHB 31	M	28	D	---------**I**	Naive	35 784	–	–	55	34 714	+	–
	CHB 32	F	60	D	n.d.	Naive	2190	–	–	19	14 400	+	–
	CHB 33	F	42	D	n.d.	Naive	12 539	–	–	90	31 836 000	–	+
	CHB 34	M	61	A	------**Y**---	naive	1271.09	–		30	438	+	–
	CHB 35	M	47	D	n.d.	naive	15 282	–	–	41	4456	–	+
NUC resolved	NUC-RES 1	M	81	n.d.		–	–	33	–	14	–	+	n.d.
	NUC-RES 2	M	57	D		–	–	26	–	28	–	+	n.d.
	NUC-RES 3	M	38	D		–	–	190	–	19	–	+	+
	NUC-RES 4	F	46	F		–	–	173	–	20	–	+	+
	NUC-RES 5	M	64	A		–	–	325	–	23	–	+	n.d.
	NUC-RES 6	M	79	D		–	–	4	–	19	–	+	n.d.
	NUC-RES 7	M	43	A		–	–	506	–	17	–	+	+
	NUC-RES 8	M	60	n.d.		–	–	2	–	31	–	+	–
	NUC-RES 9	M	36	n.d.		–	–	216.91	–	23	–	+	+
	NUC-RES 10	M	48	n.d.		–	–	>1000	–	29	–	+	–
	NUC-RES 11	M	65	n.d.		–	–	n.d.	–	32	–	+	+
	NUC-RES 12	M	65	D		–	–	2	–	19	–	+	+
	NUC-RES 13	M	62	n.d.		–	–	189.55	–	35	–	–	+
Spontaneous Seroconversion	SS 1	M	59	n.d.		–	–	45	–	30	–	+	–
	SS 2	F	69	n.d.		–	–	66	–	26	–	+	+
	SS 3	M	49	n.d.		–	–	+	–	36	–	+	n.d.
	SS 4	M	60	n.d.		–	–	270	–	31	–	+	n.d.
	SS 5	F	73	n.d.		–	–	7	–	15	–	+	n.d.
	SS 6	F	41	n.d.		–	–	451	–	11	–	+	n.d.
	SS 7	M	41	n.d.		–	–	+	–	20	–	+	n.d.
	SS 8	M	38	n.d.		–	–	n.d.	–	24	–	+	+
	SS 9	M	58	n.d.		–	–	47	–	48	–	+	n.d.
	SS 10	F	59	n.d.		–	–	+	–	12	–	+	+
	SS 11	M	74	n.d.		–	–	3	–	15	–	+	+
	SS 12	M	60	n.d.		–	–	59	–	12	–	+	–
	SS 13	F	59	D		–	–	20.11	–	16	–	+	–
	SS 14	F	50	D		–	–	2	–	36	–	+	n.d.
	SS 15	F	67	n.d.		–	–	11.2	–	19	–	+	–
	SS 16	M	69	n.d.		–	–	70.67	–	19	–	–	+
Healthy	H1	F		–		–	–	–	–	n.d.	–	Influenza matrix	
	H2	M		–		–	–	–	–	n.d.	–	Influenza matrix	
	H3	F		–		–	–	–	–	n.d.	–	Influenza matrix	
	H4	F		–		–	–	–	–	n.d.	–	Influenza matrix	
	H5	F		–		–	–	–	–	n.d.	–	Influenza matrix	
	H6	F		–		–	–	–	–	n.d.	–	Influenza matrix	
	H7	M		–		–	–	–	–	n.d.	–	Influenza matrix	

Horizontal dashed line (–) indicates a wild-type residue, whereas amino acid substitutions.

*Patient followed for >10 years; CHB diagnosed by liver biopsy; fluctuations of ALT (from normal to slightly elevated) and viraemia (from 5000 IU/mL to 40 000 IU/mL) levels; recent detection of anti-HBs combined with HBsAg.

CHB, chronic hepatitis B; HBV, hepatitis B virus; n.d, not done; SS, spontaneous seroconversion.

Seven healthy subjects (H) were enrolled as controls.

Additional 24 HLA-A2 negative treatment-naïve CHB patients were enrolled and studied to further validate the association between phenotype of total CD8 T cells and T cell responsiveness to modulatory compounds (table 2).

**Table 2:** Demographic and clinical details of HLA-A2- CHB patients

	Patient ID	Gender	Age (years)	Genotype	Therapy	HBsAg (IU/mL)	Anti HBs (UI/mL)	HBeAg (IU/mL)	ALT (IU/L)	HBV-DNA (UI/mL)
CHB	CHB 36	M	63	D	Naive	996	–	–	58	76 222
	CHB 37	F	67	A	Naive	15 970	–	–	42	9856
	CHB 38	M	20	n.a	Naive	3761	–	–	24	246
	CHB 39	F	42	D	Naive	7921	–	–	42	121 774
	CHB 40	F	52	n.a	Naive	13 945	–	–	86	1 848 322
	CHB 41	M	43	D	Naive	5459	–	–	113	1 180 000
	CHB 42	M	48	D	Naive	17 589	–	–	50	3 880 200
	CHB 43	M	81	n.a	Naive	11 695	–	–	116	2 866 690
	CHB 44	M	37	D	Naive	182	–	–	27	11 995
	CHB 45	F	44	D	Naive	691	–	–	31	10 120
	CHB 46	M	37	D	Naive	65 626	–	–	26	169 601
	CHB 47	F	61	D	Naive	3865	–	–	142	234 140
	CHB 48	M	54	D	Naive	18 436	–	–	55	1 890 237
	CHB 49	M	50	C	Naive	2028	–	–	222	12 030 348
	CHB 50	M	50	D	Naive	11 557	–	–	24	12 102
	CHB 51	M	36	D	Naive	7458	–	–	25	47 946
	CHB 52	F	29	D	Naive	30 606	–	+	59	1,35E+08
	CHB 53	M	23	D	Naive	9329	–	–	26	5837
	CHB 54	M	59	D	Naive	55 988	–	+	72	3,49E+08
	CHB 55	F	71	D	Naive	231	–	–	25	280 155
	CHB 56	F	46	D	Naive	662	–	–	19	3140
	CHB 57	M	64	D	Naive	228	–	–	19	116 997
	CHB 58	M	29	n.a	Naive	1223	–	–	54	2050
	CHB 59	M	47	D	Naive	8181	–	–	212	150 300

CHB, chronic hepatitis B; HBV, hepatitis B virus; n.a, not available.

### Phenotypic analysis of HBV-specific CD8 T cells

To define the stage of CD8+T cell differentiation, dextramer+CD8+ cells were stained with antibodies to cytokine receptors (CD127), anti-apoptosis/cell survival (Bcl-2 and TCF-1) and T cell exhaustion (PD-1, TOX and CD39) markers, as reported in online supplemental methods.

### Ex vivo functional assessment of HBV-specific CD8 T cells

Intracellular cytokine staining (ICS) for IFN-γ and TNF-α was performed on dextramer^+^CD8+ T cells following stimulation with phorbol-12-myristate-13-acetate (PMA) (100 ng/mL) and ionomycin (1 µg/mL) for 4 hours (see online supplemental methods).

### ‘z-Score’ and ‘Exhaustion index*’*

Median fluorescence intensity (MdFI) values of exhaustion and memory markers (PD-1, TOX, CD39 and CD127, Bcl-2, TCF-1, respectively) expressed by HBV-specific CD8 T cells were standardised by calculation of the z-Score (details in online supplemental materials) to overcome the problem raised by the different order of magnitude of MdFI values detected for individual parameters. All z-Scores of each patient’s MdFI were calculated relative to reference populations (original set of data from which m and s were derived) represented by NUC-RES and SS. A new variable, named Exhaustion Index (EI), was then created by averaging PD-1, TOX, CD39, CD127, Bcl-2 z-Scores: EI=mean (z-PD-1 + z TOX + z-CD39 – z-CD127 – z-Bcl-2). This new variable takes into account the contribution of each MdFI value irrespective of its original scale. A threshold of 2 (like in the gaussian case) was choosen as cut-off to separate patients with high vs low EI.

### In vitro T-cell expansion and treatment with immunomodulators

T cell cytokines (IFN-γ, TNF-α) were tested by intracellular cytokine staining (ICS), as previously described^18^ on short-term T-cell lines treated with the following compounds (see online supplemental materials):

- Trans-Resveratrol.
- MitoTEMPO.
- Anti-programmed cell death ligand 1 (PD-L1).
- Small molecule PD-L1 inhibitor.

### Direct sequencing analysis

The HBV core genome was sequenced in 29 patients with chronic HBV infection (see details in online supplemental materials).

### Statistical methods

Two-tailed Mann-Whitney U test and Wilcoxon matched-pairs test were used for statistical comparisons. Differences between multiple patient groups were evaluated by the Kruskal-Wallis non-parametric test corrected for pairwise multiple comparisons. The Spearman’s rank-correlation test was applied for correlation analysis. Further details in online supplemental materials.

## Results

### Phenotypic heterogeneity of HBV-specific CD8 T cells in CHB

HLA-A02-restricted core_18-27_-specific CD8+T cells were analysed ex vivo in 35 CHB patients before starting antiviral therapy, all with stably elevated or fluctuating viraemia and ALT levels (as typically observed in HBeAg- CHB patients), in 13 NUC treated patients after anti-HBs seroconversion and therapy suspension (NUC-RES) and in 16 subjects who became anti-HBs+following chronic HBV carriage (‘SS’) (table 1 and figure 1A).

Flow cytometry analysis of the exhaustion and memory markers PD-1, TOX, CD39, CD127, Bcl-2 and TCF1^4 6 7 19^ showed higher PD-1, TOX, CD39 and lower Bcl-2, CD127 expression on core_18-27_-specific CD8 T cells from untreated CHB patients compared with resolved subjects (NUC-RES and SS) and to influenza-specific CD8 T cells from healthy controls. Instead, TCF1 expression was similar in all patient categories (figure 1B,C). A direct correlation was observed in CHB patients between individual exhaustion markers (PD-1 vs TOX, CD39 vs TOX and CD39 vs PD-1) on one hand and between individual differentiation/memory markers (Bcl-2 vs TCF1 and CD127 vs Bcl-2) on the other (figure 1D). Instead, exhaustion and differentiation/memory markers were inversely correlated to each other (figure 1D).

**Figure 2:**
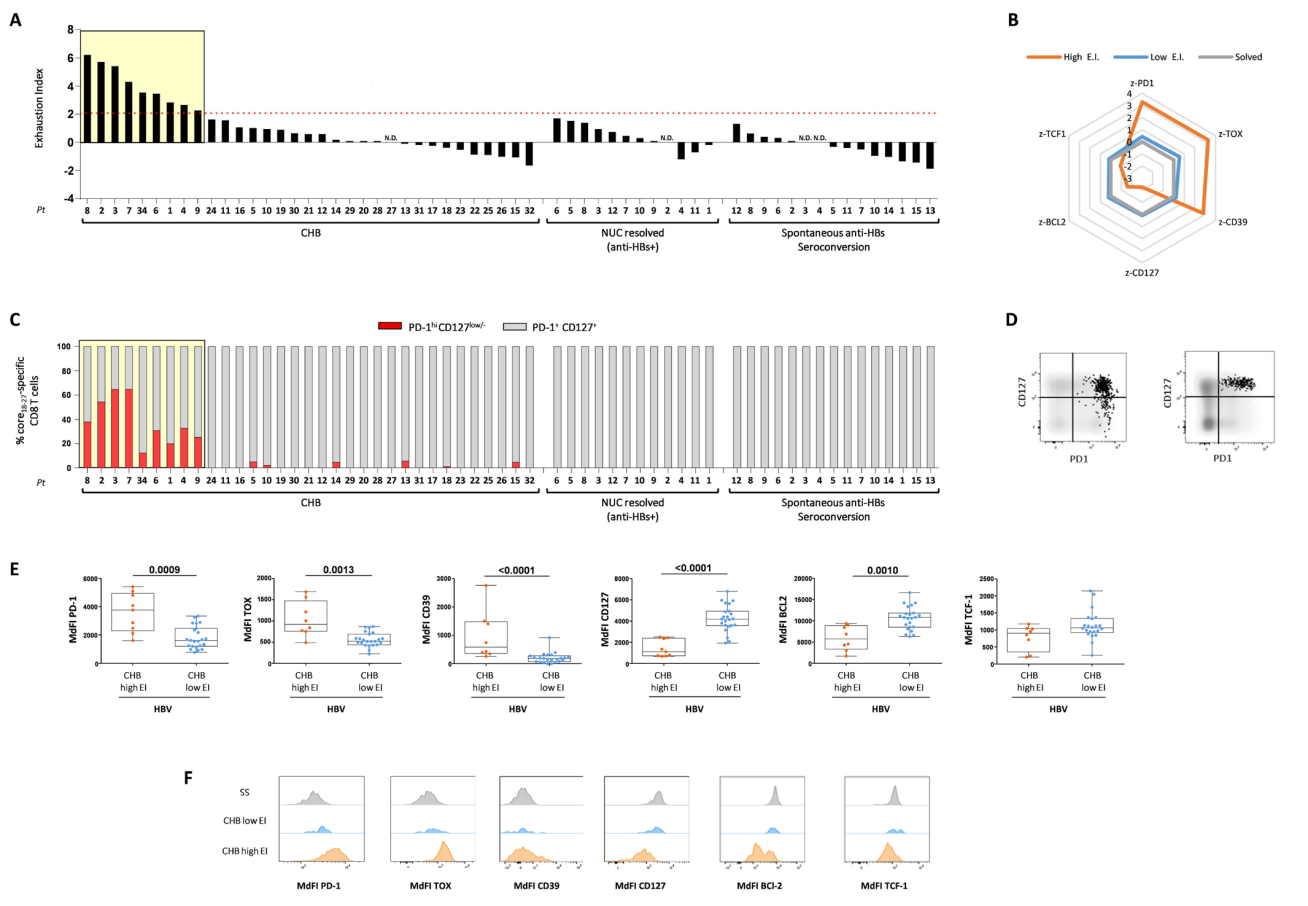
Classification of CHB patients according to CD8 T cells expression of exhaustion and memory/differentiation markers. (A) EI values were defined by PD-1, CD39, TOX, CD127, Bcl-2 and TCF1 expression on core_18-27_-specific CD8 T cells for each study cohort. Each bar represents an individual patient. The yellow area indicates patients with EI values above 2. (B) Radar plots depict the mean z-Score values of the indicated HBV-specific CD8 T cell exhaustion and memory/differentiation markers. Blue and orange lines indicate z-Score values of patients with high or low EI values, respectively; the grey line NUC-induced and spontaneous anti-HBs seroconverters. (C) Frequencies of PD-1^high^CD127^low/-^ and CD127+/PD-1+ cells among the core_18-27_-specific CD8 T cell population (red and grey bars, respectively). Each bar represents an individual patient. (D) Representative FACS plots from two chronic patients with or without the PD-1^high^CD127^low/-^ subset. (E) PD-1, TOX, CD39, CD127, Bcl-2 and TCF1 expression on core_18-27_-specific CD8 T cells of CHB patients with high and low EI values. Box-whisker plots as in figure 1; each dot represents a single patient. Statistics by the Mann-Whitney U test. (F) Representative histogram plots for each parameter in each patient cohort. CHB, chronic hepatitis B; EI, Exhaustion Index; HBV, hepatitis B virus.

Quantification of the overall level of CD8 T cell exhaustion in each patient by an exhaustion score (EI) that we generated from z-standardised MdFI values of PD-1, TOX, CD39, CD127, Bcl-2, allowed to distinguish two cohorts of CHB patients with EI values lower or greater than 2, comprising 72% (23 patients; figure 2A) and 28% (9 patients; figure 2A, black bars within the yellow square) of the overall CHB cohort, respectively. Combined analysis of the different exhaustion and memory markers is well recapitulated by the radar plot in figure 2B, where blue and orange lines correspond to each z-normalised phenotypic parameter in CHB patients with high and low EI values, respectively. The high EI CHB patient group was also characterised by the presence of a unique CD8 T cell subset composed of PD-1^high^CD127^low/-^ cells that was poorly represented or totally negative in the remaining group of CHB patients and completely absent in the resolver groups (NUC-RES and SS) (figure 2C,D). As depicted also in figure 2B, a significant difference in the expression of PD-1, TOX, CD39, Bcl-2 and CD127 was detected in core_18-27_-specific CD8 T cells of CHB patients with high and low EI (figure 2E,F). A statistically significant positive correlation was detected in naive CHB patients by comparing EI with ALT levels but not with viral load or HBsAg values (online supplemental figure S1).

10.1136/gutjnl-2022-327202.supp2 Supplementary data

**Figure 3:**
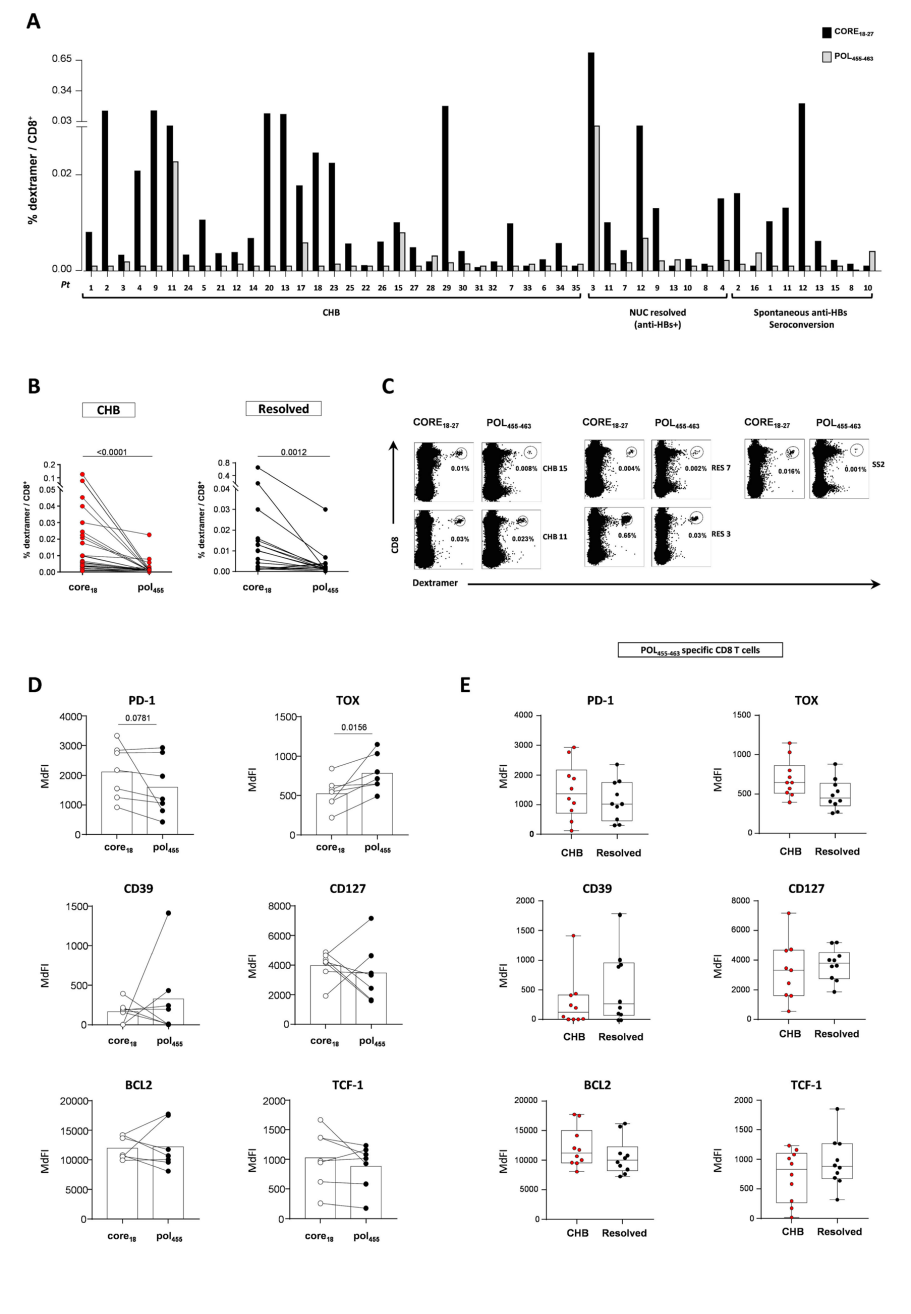
Different detection rate and phenotype of core_18-27_-specific and pol_455-463_-specific CD8 T cells. (A, B) Frequencies of core_18-27_- and pol_455-463_-specific CD8 T cells within the total CD8+T cell population from 31 CHB and 18 resolved patients who showed a detectable frequency for at least one HBV dextramer (paired core and polymerase data are illustrated in panel B). (C) Representative FACS plots from two CHB and three resolved patients. (D) PD-1, TOX, CD39, CD127, Bcl-2 and TCF1 expression within core_18-27_- and pol_455-463_-specific CD8 T cells from 7 CHB patients; statistics by Wilcoxon matched-pairs test. (E) PD-1, TOX, CD39, CD127, Bcl-2 and TCF1 expression on pol_455-463_-specific CD8 T cells in CHB and resolved patients (n=10 for both groups). CHB, chronic hepatitis B; HBV, hepatitis B virus.

In order to extend T cell analysis to another HBV antigen, CD8 T cells from patients with active (n=45) and resolved (n=26) chronic infections were also stained with the pol_455-463_ dextramer. Pol_455-463_-specific CD8 T cells were found only in a limited proportion of the HBV-infected chronic patients (11 of 45 tested, 24%) (table 1 and figure 3A). In all patient categories they were significantly less represented than core_18-27_-specific CD8 T cells (figure 3B,C, p<0.0001 and p=0.0012 in CHB and resolved patients, respectively). In line with previous studies,^14–16^ in chronic patients pol_455-463_-specific CD8+T cells expressed higher levels of TOX and lower levels of PD-1 (figure 3D, p=0.0156 and p=0.07, respectively) compared with core_18-27_-specific CD8+T cells, while no differences in CD39, Bcl-2, TCF1 and CD127 expression were observed between CD8 cells of different specificity (figure 3D). No significant differences were also detected in the expression of exhaustion and differentiation/memory markers between pol_455-463_-specific CD8+T cells from HBV-infected chronic patients and individuals with spontaneous or NUC-induced control of infection (figure 3E). Overall, our results suggest that pol_455-463_ and core_18-27_-specific CD8 T cells in CHB significantly differ with respect to peripheral blood frequencies and phenotypic parameters highlighting two distinct exhaustion profiles.

### Different CD8 T cell functionality of CHB patients with different EI values

**Figure 4:**
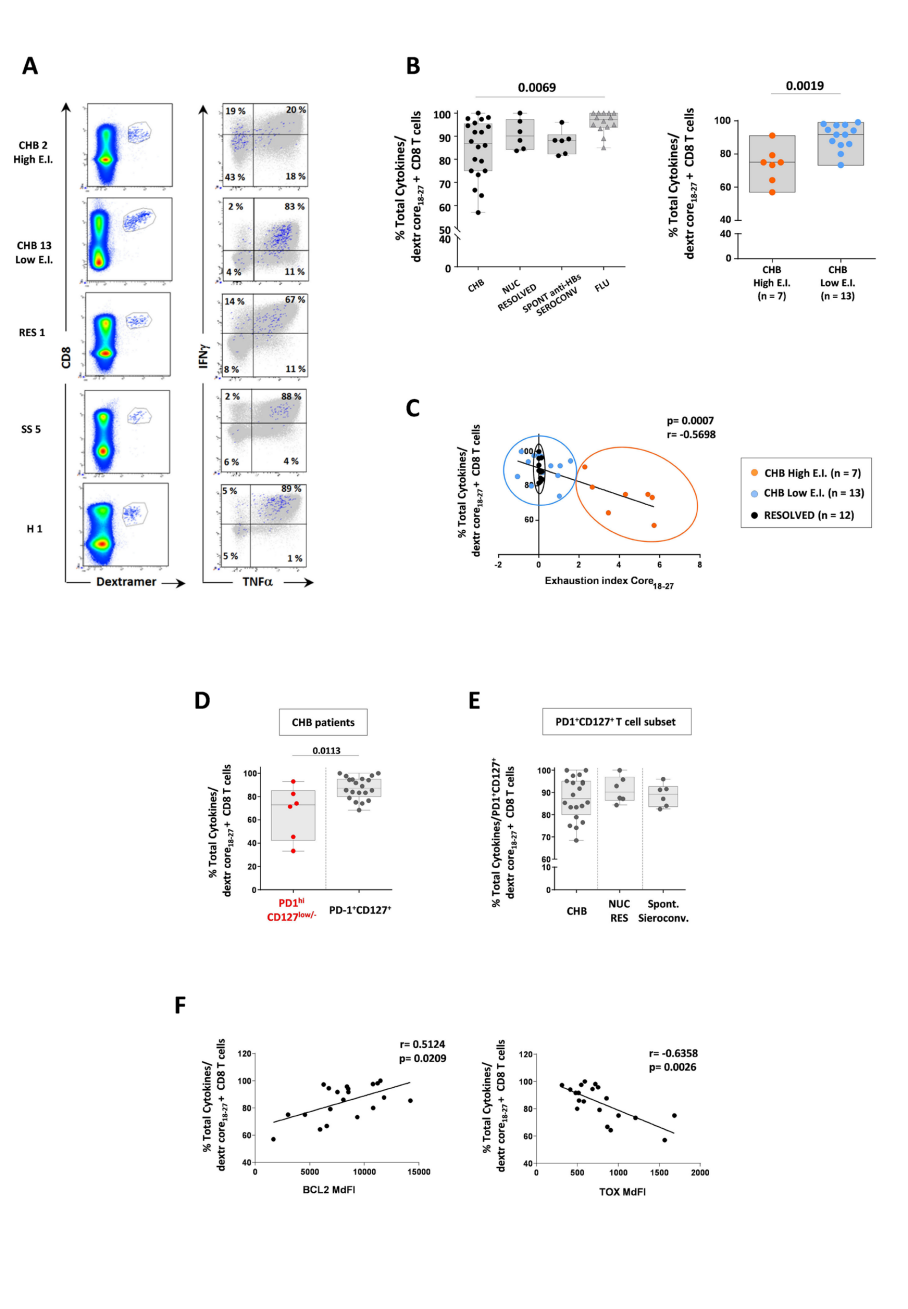
Ex vivo HBV-specific T cell functional analysis. Cytokine production by HBV-specific and influenza-specific CD8 T cells: (A) representative plots; (B) different patient categories assessed after PBMC stimulation with PMA and Ionomycin (box whisker plots, on the left); CHB patients split according to EI values (right graph). (C) Correlation between cytokine production by the core_18-27_-specific CD8 T cells and EI in chronic patients with high (orange dots) and low (blue dots) EI, respectively, and in HBV resolved patients (black dots). (D, E) Cytokine production by PD-1^high^CD127^low/-^ and PD-1^+^CD127^+^ HBV-specific CD8 T cells from CHB patients and from PD-1^+^CD127^+^ CD8 T cells from the different patient categories. (F) Correlation between cytokine production and Bcl-2 (left graph) or TOX (right graph) expression by core_18-27_-specific CD8 T cells. Statistics by the Kruskal-Wallis with Dunn’s correction test (B, E) and the Spearman’s correlation test (C, F). CHB, chronic hepatitis B; EI, Exhaustion Index; HBV, hepatitis B virus; NUC RES, NUC resolved patients; PBMC, peripheral blood mononuclear cell; SS, spontaneous seroconversion.

Cytokine production by core_18-27_-specific CD8 T cells was then analysed ex vivo (figure 4A). The same analysis on pol-specific CD8 T cells was precluded by their extremely low frequency. Functionality of core-specific CD8 T cell was very variable, because some CHB patients showed cytokine production levels comparable to NUC and spontaneous anti-HBs seroconverters, while other CHB patients produced cytokines less efficiently (figure 4B, left graph).

HBV-specific CD8 T cells from CHB patients with lower EI were more efficient in cytokine production (figure 4B, right graph) which was inversely correlated to the EI (figure 4C). PD-1^high^CD127^low/-^ CD8 T cells were functionally less efficient than PD-1^+^CD127^+^ cells (p=0.0113, figure 4D). A functional comparison of the PD-1^high^CD127^low/-^ CD8 T cell subset in the different patient cohorts was not possible because this subset was selectively present only in CHB patients. Instead, PD-1^+^CD127^+^ CD8 T cells were detectable in all patient cohorts and their cytokine production was variable in chronic patients, some of whom were less efficient than NUC and spontaneous resolvers (figure 4E).

Total cytokine production by the overall HBV-specific CD8 T cell population showed a positive and negative correlation with Bcl-2 and TOX levels, respectively (figure 4F). The same trend (but without significant levels of correlation) was observed with the other exhaustion (PD-1 and CD39) and memory (CD127, TCF-1) markers.

### CD8 T cell phenotypic profile and response to immune modulatory interventions in vitro

We then assessed whether HBV-specific CD8 T cells of CHB patients with different EI levels, were more or less sensitive to the effect in vitro of antioxidant, polyphenolic and PD-1/PD-L1 targeting compounds^18 20–26^ and a selective toll-like receptor 8 (TLR8) agonist.^27 28^

**Figure 5:**
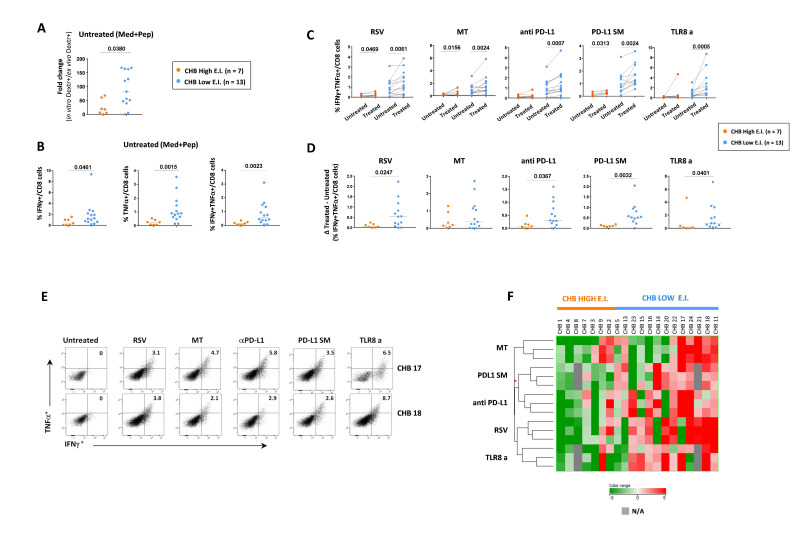
Effect of immune modulatory interventions on the HBV-specific CD8 T cell function. Expansion capacity calculated as the ratio (fold-change) between in vitro and ex vivo frequencies of core_18-27_ dextramer^+^ CD8 T cells (A) and percentage of cytokine-positive CD8 T cells (B) in short-term T-cell lines generated by core_18-27_ peptide stimulation of PBMC from CHB patients with high (n=7) and low (n=13) EI; statistics by Mann-Whitney U test. (C) Percentage of double-positive IFNγ+TNFα+ CD8 T cells in paired short-term T-cell lines generated as in (B) in the presence (treated) or absence (untreated) of Resveratrol (RSV), MitoTempo (MT), anti-PD-L1, a small PD-L1 inhibitor molecule (PD-L1 SM) and a TLR8 agonist (TLR8a) from chronic patients with high (n=7) and low (n=13) EI; statistics by the Wilcoxon-matched-paired test. (D) Delta values of double-positive IFNγ+TNFα+ CD8 T cells derived by subtracting CD8 T cell frequencies of untreated from treated short-term T-cell lines (black lines indicate the median values; statistics by Mann-Whitney U test). (E) Representative examples of cytokine production in two CHB patients. (F) Hierarchical-clustering of HBV-specific CD8 T-cell responses induced by in vitro core_18-27_ stimulation in presence of the different treatments in chronic patients with high (n=7 orange) and low (n=13 blue) EI. CHB, chronic hepatitis B; EI, Exhaustion Index; HBV, hepatitis B virus; N/A, not available; PBMC, peripheral blood mononuclear cell.

In the absence of these immune modulatory compounds, T-cell lines produced in CHB patients with low EI showed better expansion and cytokine production compared with T cell lines from patients with high EI (figure 5A,B), in keeping with ex vivo results.

All tested compounds had significantly greater effect on HBV-specific CD8 T cells of patients with low EI (figure 5C–E, online supplemental figures S2, S3). Functional changes induced by these immune modulatory compounds are well recapitulated by hierarchical-clustering analysis, which demonstrates a clear segregation of upregulated and downregulated functional parameters in chronic patients with low and high EI (figure 5F). Interestingly, the anti-PD-L1 monoclonal antibody and the PD-L1 small molecule inhibitor^24^ displayed a common clustering indicating similar quality and intensity of CD8 T cell modulation. Likewise, the effects of Resveratrol and the TLR8-agonist clustered together, suggesting a comparable pattern of modulatory activity (figure 5F). By direct comparison of the different compounds, the TLR8-agonist was the most effective in the reconstitution of the CD8 T cell function (online supplemental figure S4).

10.1136/gutjnl-2022-327202.supp3 Supplementary data

10.1136/gutjnl-2022-327202.supp4 Supplementary data

10.1136/gutjnl-2022-327202.supp5 Supplementary data

### Prediction of immune T cell reconstitution by phenotypic T cell analysis of the whole CD8 T cell population

**Figure 6:**
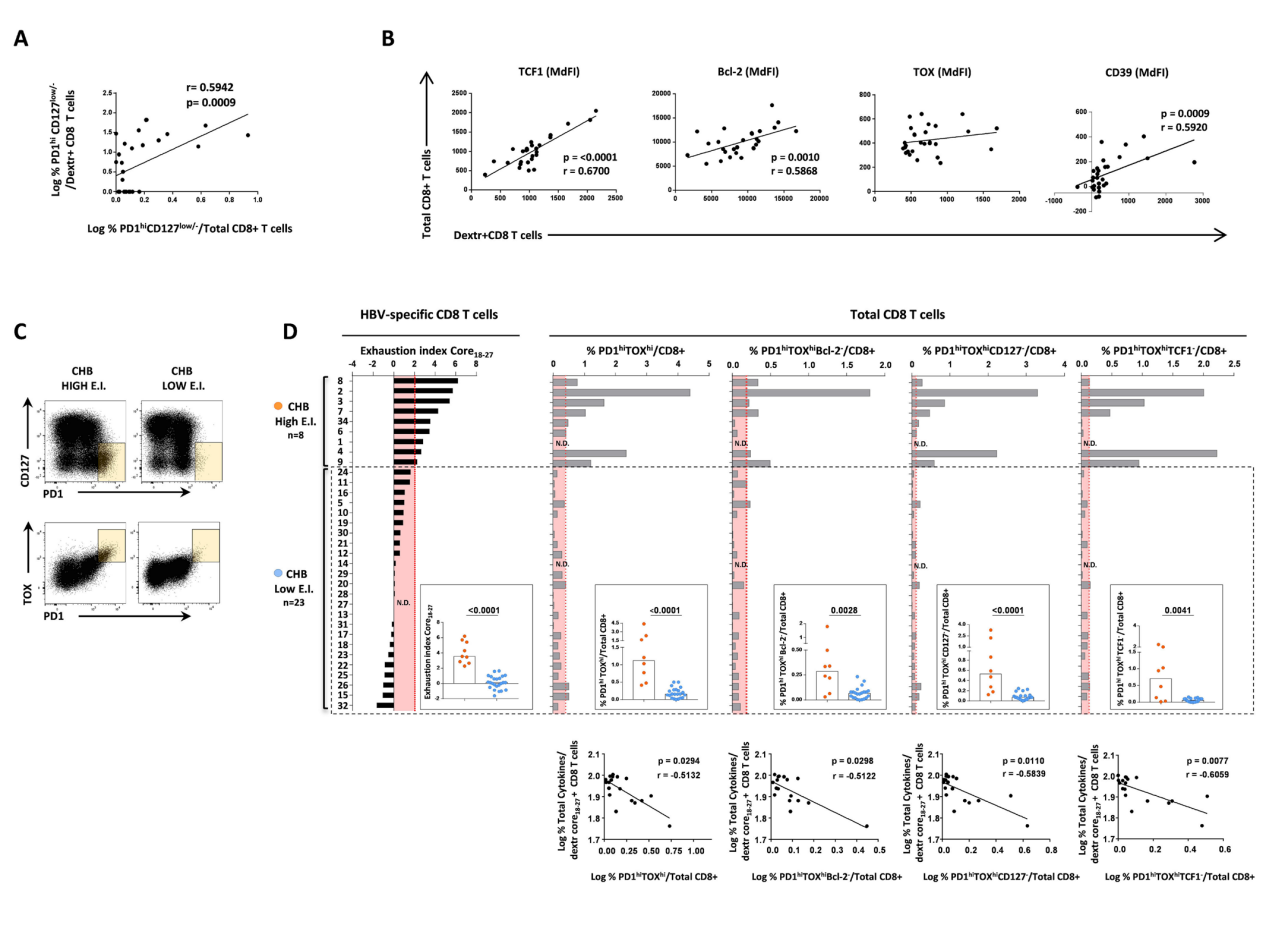
Phenotypic analysis of the total CD8 T cell population. (A) Correlation between logarithmic frequency values of PD-1^high^CD127^low/-^ T cells among core_18-27_-specific and total CD8 T cells. (B) Correlations of TCF1, Bcl-2, TOX and CD39 expression in total and HBV-specific CD8 T cells. (C) Representative plots of PD-1^high^/CD127^low/-^ and TOX^+^/PD-1^high^ CD8 T cell subsets (yellow boxes) in two CHB patients with high and low EI. (D) Top: phenotypic profiles of total CD8 T cells of individual patients (grey bars) in relation to the corresponding EI values derived from HBV-specific CD8 T cell analysis (black bars, on the left). Red areas indicate the significance threshold for each phenotypic CD8 T cell subpopulation calculated by using a ROC curve analysis based on exhaustion index classification criteria (details in online supplemental materials). In the internal squares, each dot represents the frequency of total CD8 T cells expressing the indicated phenotypic profile in chronic patients with high or low EI (Mann-Whitney test). Bottom: correlation between frequency of cytokine positive core_18-27_-specific CD8 T cells ex vivo and frequency of PD-1^high^/TOX^high^, PD-1^high^/TOX^high^/ Bcl-2^-^, PD-1^high^/TOX^high^/CD127^-^ or PD-1^high^/TOX^high^/TCF1- among total CD8 T cells in CHB patients. Statistics by the Spearman’s correlation test (A, B, D). CHB, chronic hepatitis B; EI, Exhaustion Index; HBV, hepatitis B virus; ROC, receiver operating characteristic.

Evidence of CD8 T cell rejuvenation by immune modulatory treatment in vitro and prediction of response to immune modulation are based on the analysis of a single antigenic specificity and may thus be partially relevant with respect to the multispecific T cell response primed in vivo by infection. Interestingly, we noted a significant positive correlation between core_18-27_-specific and total CD8 T cells relative to the frequency of PD-1^high^CD127^low/-^ cells (figure 6A) and to the expression of differentiation/exhaustion markers, such as Bcl-2 and CD39, and transcription factors, such as TCF-1 and TOX (figure 6B). This evidence prompted us to assess whether the identification of patients most likely to respond to immunomodulatory compounds in vitro can be accomplished also by phenotypic analysis of total CD8 T cells.

Remarkably, the same distribution of patients derived from the calculation of the EI in HBV-specific CD8 T cells, was also found by analysing the expression of different combinations of PD-1, CD127, Bcl-2 and TOX on total CD8 T cells (figure 6C,D). In particular, the frequency of PD-1^high^TOX^high^, PD-1^high^TOX^high^Bcl-2^-^, PD-1^high^TOX^high^TCF1^-^ and PD-1^high^TOX^high^CD127^-^ subsets in total CD8 T cells was significantly greater in CHB patients with high EI (details in online supplemental results and figure S5). These observations indicate that total CD8 T cell phenotyping can reflect HBV-specific CD8 T cell analysis for the identification of patient cohorts with different antiviral function and different in vitro responsiveness to modulatory compounds. This conclusion is further supported by the significant inverse correlation between cytokine production by HBV-specific T cells and the phenotypic profile of total CD8 T cells (figure 6D, bottom charts) and by the significant inverse correlation between HBV-specific T cell responses to the distinct immune modulations and the frequency of PD-1^high^TOX^high^, PD-1^high^TOX^high^Bcl-2-, PD-1^high^TOX^high^CD127- and PD1^high^TOX^high^TCF1^-^ CD8 T cell subsets (online supplemental figure S6). Instead, significant correlations between phenotypes of the bulk CD8+T cell population and clinical/virological parameters (HBV-DNA, ALT, HBsAg) were not observed in treatment naïve CHB patients, with the exception of a positive trend observed only for ALT levels (online supplemental figure S7).

10.1136/gutjnl-2022-327202.supp6 Supplementary data

10.1136/gutjnl-2022-327202.supp7 Supplementary data

10.1136/gutjnl-2022-327202.supp8 Supplementary data

**Figure 7:**
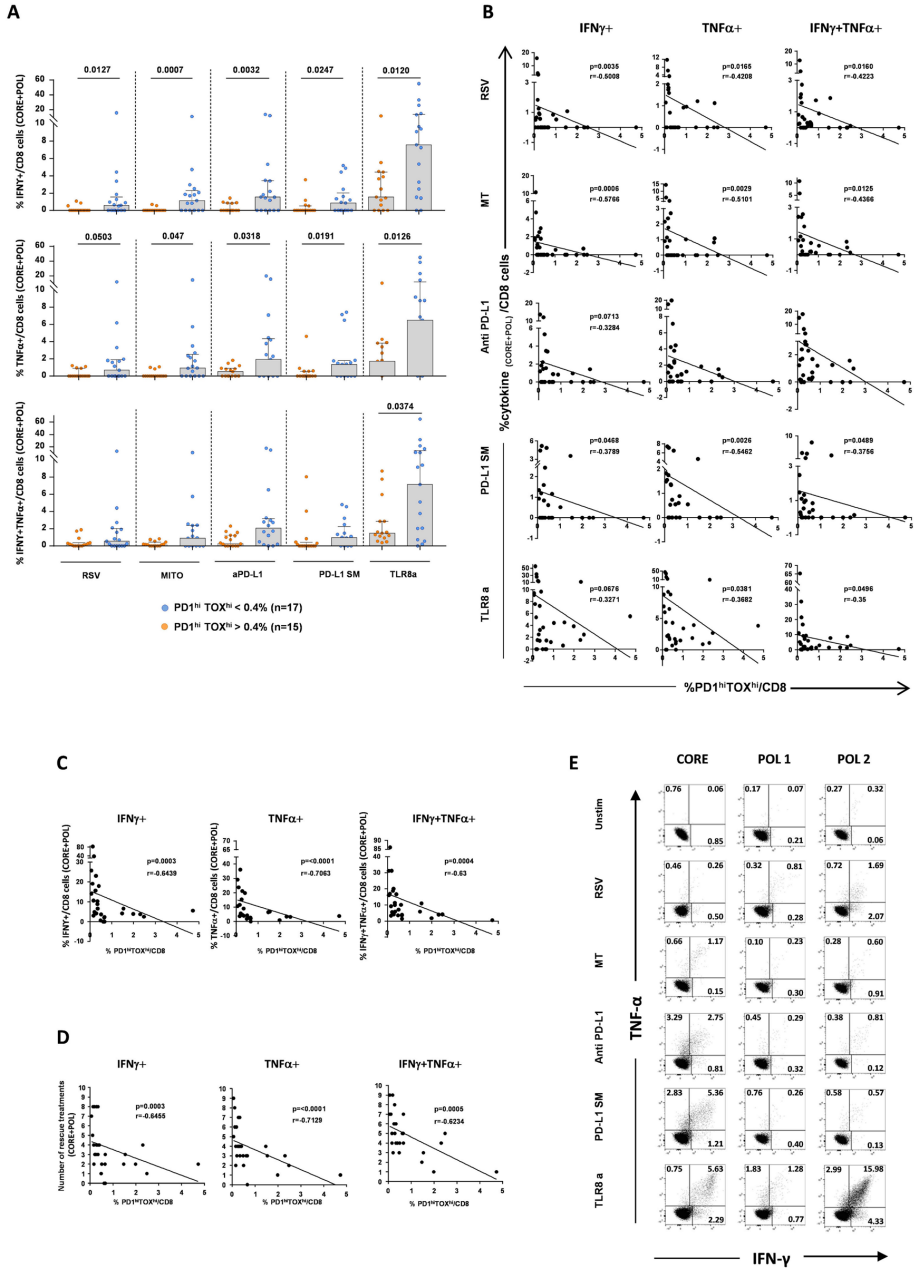
CD8 T cell phenotype can predict recovery of multi-specific T cell responses to immune modulation. Delta values of IFNγ+, TNFα+ and double-positive IFNγ+TNFα+ CD8 T cells derived by subtracting CD8 T cell frequencies of untreated from treated short-term T-cell lines generated by stimulation of PBMC from CHB patients (n=32) with 15-mer overlapping HBV core and polymerase peptide pools. Data are shown as sum of T cell responses against the two antigens. (A) Segregation of individual CD8 T cell responses to core and polymerase peptides based on the PD-1^high^TOX^high^ CD8 T cell frequency threshold (0.4% value) previously obtained using an ROC curve analysis as illustrated in figure 6D. Statistics by the Mann-Whitney U test. (B–D) Correlation between PD-1^high^TOX^high^ CD8 T cell subset frequency and percentage of IFNγ+, TNFα+ and double-positive IFNγ+TNFα+ CD8 T cells in short-term T-cell lines generated as described above, illustrated for each immune modulation (B), as cumulative data of all treatments (C) and as numbers of immune modulatory agents able to induce a positive response (D). Statistics by the Spearman’s correlation test. (E) Representative examples of cytokine production in a CHB patient with low PD-1^high^TOX^high^ CD8 T cell frequency. CHB, chronic hepatitis B; HBV, hepatitis B virus; PBMC, peripheral blood mononuclear cell.

We then attempted to validate the T cell functional restoration effect of the immune modulatory compounds and the predictive value of the CD8 T cell-based phenotypic score in the context of a multispecific CD8 T cell response stimulated with broader peptide panels covering the entire core and polymerase sequences, which obviously reflect more closely the complexity of the immune response induced by infection.

The validation cohort was composed of additional 32 newly enrolled treatment naïve CHB patients (24 and 6 HLA-A2 negative and positive, respectively, details in tables 1 and 2). Levels of cytokine production were assessed upon PBMC stimulation with 15-mer overlapping peptide pools covering HBV core and polymerase antigens in the presence or absence of the different immune modulatory agents. A frequency threshold of 0.4% PD-1^high^TOX^high^ CD8 T cells among the overall CD8 T cell population, defined by an ROC curve analysis of phenotypic data generated in HLA-A2 positive patients on core_18-27_-specific and total CD8 T cells and shown to distinguish patients with different levels of EI values, was applied to analyse the new data set. Results confirm that CHB patients with a PD-1^high^TOX^high^ CD8 T cell frequency below 0.4% show a better response to immune modulatory compounds also when CD8 T cells are stimulated with peptide pools corresponding to the overall core and polymerase sequences able to stimulate a multispecific T cell response (figure 7A). In line with this observation, a statistically significant inverse correlation was observed between cytokine production by core and polymerase stimulated CD8 T cells and PD-1^high^TOX^high^ CD8 T cell subset frequency (figure 7B–E). Although both core-specific and polymerase-specific CD8 T cell responses were improved by in vitro modulation, a slightly greater contribution to the overall HBV-specific T-cell restoration was given by core-specific responses (online supplemental figure S8).

10.1136/gutjnl-2022-327202.supp9 Supplementary data

**Figure 8:**
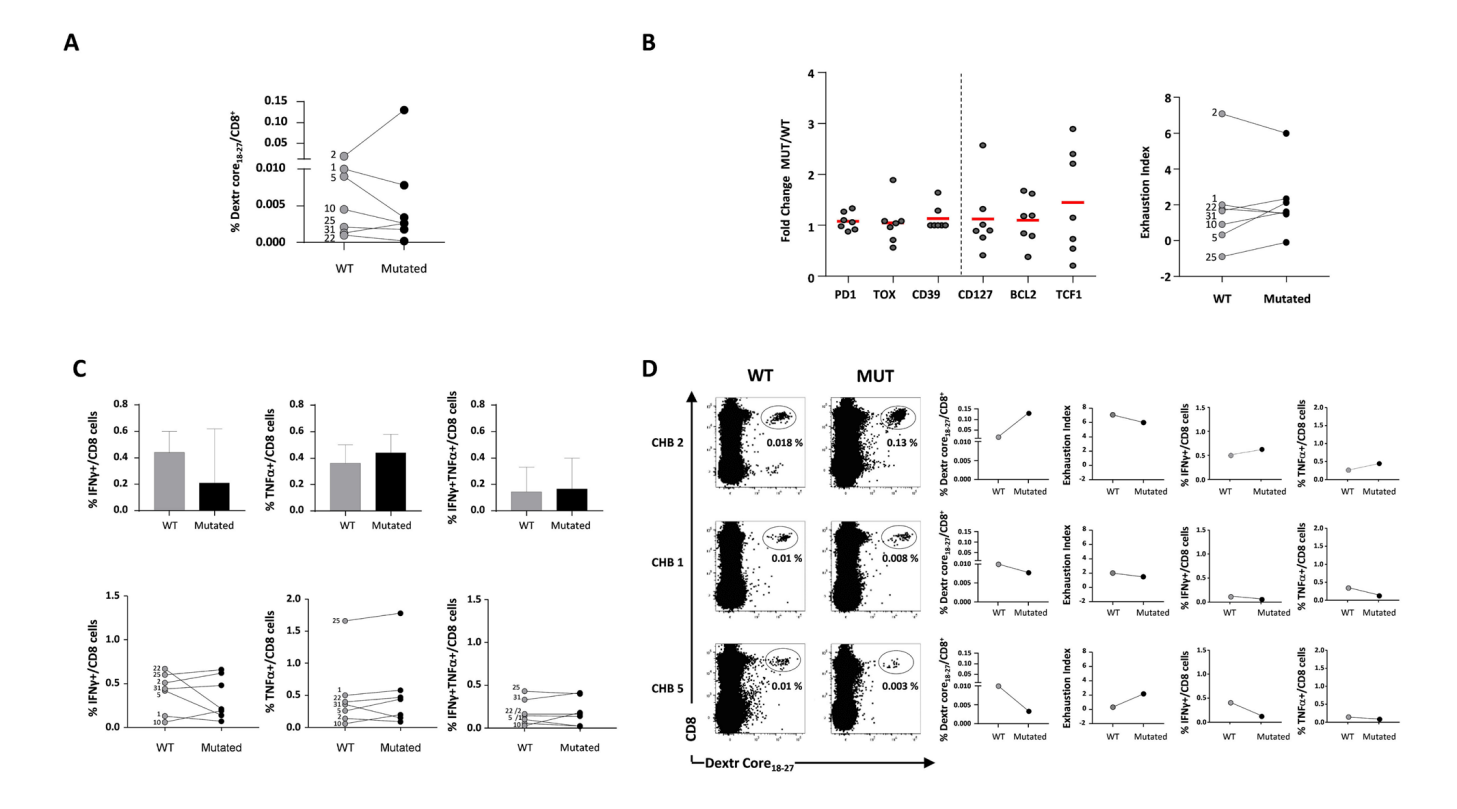
Impact of mutations on core_18-27_-specific CD8 T cell responses. (A) Ex vivo frequency of wild-type-specific or variant-specific core_18-27_ CD8 T cells by staining with the corresponding wild-type of mutated peptide HLA class I dextramers from seven CHB patients; next to each dot, the CHB patient ID number is indicated; statistics by Wilcoxon matched-pairs test. (B) Ratio (fold-change) between the MdFI values of the indicated phenotypic markers expressed by CD8 T cells targeting the mutated or the wild-type core_18-27_ epitopes (left graph) and EI values defined by PD-1, CD39, TOX, CD127, Bcl-2 and TCF1 expression (right graph). (C) Percentage of cytokine-positive CD8 T cells in short-term T-cell lines generated by WT or mutated core_18-27_ peptide stimulation of PBMCs from seven CHB patients. The column chart represents median values (upper), while the dot graph shows individual patient responses to wild-type and mutated peptides (lower); statistics by the Wilcoxon-signed-rank test. (D) Dot-plots of ex vivo frequencies of wild-type- or mutated-specific core_18-27_ CD8 T cells in CHB patients 2, 5, 1. On the right, the corresponding graphs illustrating frequency, phenotype (EI) and function are shown. CHB, chronic hepatitis B; EI, Exhaustion Index; MdFI, median fluorescence intensity; PBMC, peripheral blood mononuclear cell.

These experiments confirm the results obtained with HBV-specific CD8 T cells of a single specificity and validate in the context of a multispecific CD8 T cell response the feasibility of distinguishing patients more prone to respond in vitro to immune modulation by a simple phenotypic assessment of CD8 exhaustion parameters.

### Impact of viral sequence variation on phenotype and function of core_18-27_-specific CD8 T cells

Since HBV is a highly variable virus we investigated whether mutations within the core_18-27_ sequence can contribute to the functional heterogeneity of HBV-specific CD8 T cell responses. The HBV core genome was sequenced in 29 patients with chronic HBV infection (table 1). Notably, a wide sequence variation with a high mutation rate was detected in the HLA-A2 restricted core_18-27_ epitope in CHB viraemic patients, because mutated core sequences were detected in 73% of the analysed epitopes (21 patients out of 29). The effect of mutations was quite variable, although frequency, phenotype and function of core_18-27_-specific CD8 T cells did not change significantly in most of the patients. For example, only CHB 2 and 5 showed more evident frequency variations in opposite directions (figure 8A). However, their phenotypic profile did not change significantly because the EI increase in patient 5 did not reach a value greater than 2 (which is the threshold discriminating high and low levels of exhaustion) (figure 8B) and the decline in IFN-γ production was not associated with a parallel and comparable decline in TNF-α production (figure 8C,D). Similarly, in patient 2 the slight decline in SI (too modest to induce a decline below the threshold of 2) (figure 8B) was associated with a very modest increase in IFN-γ and TNF-α production (figure 8C,D).

## Discussion

In chronic HBV infection HBV-specific CD8 T cells appear to be functionally heterogeneous.^2 12 13 16 29^ To elucidate further the phenotypic and functional features of this CD8 T cell heterogeneity and to identify CD8 T cell-based predictors of response to immune reconstitution therapies we first selected a homogeneous chronic active hepatitis patient population with persistent viraemia and liver inflammation, eligible for therapy and expected to harbour terminally exhausted HBV-specific CD8 T cells. This is particularly relevant because part of the data so far generated in this area are derived from heterogeneous groups of patients, frequently including also patients with an inactive infection. Particular attention was also paid to identify the rare patient populations able to achieve functional cure of chronic infection either spontaneously or after NUC therapy, which are essential to understand whether reacquisition of a fully protective antiviral activity is achievable after long-term exposure to high antigen and viral loads. Despite low frequency of HBV-specific CD8 T cells, an in-depth phenotypic and functional analysis was performed directly ex vivo.

Second, we studied the effect in vitro of antioxidant, polyphenolic, TLR8 agonist and PD-1/PD-L1 blocking compounds on phenotype and function of HBV-specific CD8 T cells to select optimal candidate targets for immune modulatory therapies. Finally, we searched for predictors of in vitro response to immune modulation, in the perspective of a possible clinical translation aimed at personalising immune therapies for CHB.

By combining the analysis of different exhaustion (PD-1, CD39, TOX) and memory (CD127, Bcl-2, TCF1) markers we identified a specific CD8 T cell phenotypic score, that we named EI, suitable to distinguish cohorts of CHB patients with different CD8 T cell exhaustion levels. By focusing on PD-1 and CD127 staining, we found that a subset of PD-1^+^CD127^+^ HBV-specific CD8 T cells was present in all patient categories, irrespective of the clinical condition and level of virus control. This is consistent with the detection of TCF1+CD127+PD-1+ memory like CD8 T cells in chronic HCV infection, that persist after therapy-induced HCV clearance and show higher levels of PD-1 and Eomes expression, as well as reduced cytokine production compared with conventional memory HCV-specific T cells generated after spontaneous resolution of HCV infection.^4 30 31^ Instead, PD-1^hi^CD127^low/-^ HBV-specific CD8 T cells were present in a more limited proportion of untreated chronic viraemic patients with higher EI levels.

Higher cytokine production was detected in CHB patients with a less exhausted phenotype, marked by low EI levels and the total lack of PD-1^hi^CD127^low/-^ HBV-specific CD8 T cells. Instead, CHB patients with higher EI levels and a greater representation of PD-1^hi^CD127^low/-^ CD8 T cells appeared to be significantly less efficient in cytokine production. All together, our observations suggest that the phenotype-based EI score actually reflects the CD8 T cell function and may thus represent a composite multiparametric score for quantification of CD8 T cell exhaustion.

Interestingly, a functional exhaustion score (FES) was also developed by Bengsch *et al* with the same predictive purpose in HIV and human cancer settings, based on the assessment of exhaustion and effector/memory functional parameters. Although Bengsch’s FES score and our EI score are not necessarily expected to be concordant because of the different clinical settings of analysis and application, their agreement would strongly support the robustness our phenotype-based EI. Preliminary analysis of IFN-γ and TNF-α production profiles (the only components of the FES score available in our dataset) does not seem, however, to show a different behaviour in patients with high or low EI, as predictable if the two scores were concordant.^32^

To the interindividual CD8 T cell heterogeneity may contribute the presence of mutations within the core_18-27_ epitope. Thus, the core 18–27 region was sequenced in 29 CHB patients and the effect of mutations on CD8 responses was assessed in 7 of them who harboured variant core_18-27_ epitopes. HBV mutations caused variable effects on HBV-specific CD8 T cells. Most of them were neutral with no effects on CD8 frequency, phenotype and function, but a few mutations caused increase or decline in CD8 T cell frequencies. These changes, however, had only a marginal impact on phenotypic and functional CD8 T cell profiles because distribution of the patients into high or low EI classes was not modified by the results obtained with mutated instead of prototype sequences. Additional studies are however warranted to drive definitive conclusions because only seven patients with mutated epitopes were analysed in this study.

The possibility to rank the level of CD8 T cell exhaustion of single chronic HBV patients may be extremely helpful for the identification of patients with immunological conditions still amenable of functional reconstitution, for example, with antioxidant and polyphenolic compounds, such as MitoQ and MitoTempo or Resveratrol and Oleuropein, that have recently been proposed by us as promising candidates for CD8 T cell-based therapeutic strategies.^18 20^ To investigate this issue, we first tested the capacity of different metabolic and immune modulatory compounds to improve HBV-specific CD8 T cell responses in our cohort of chronic viraemic patients with well characterised levels of CD8 T cell exhaustion, and, second, we looked for specific predictive parameters of response to immune modulation. In addition to mitochondrial antioxidant and polyphenolic compounds, HBV-specific CD8 T cells were treated in culture with the TLR8 agonist Selgantolimod and a novel small PD-L1 inhibitor molecule, which represents a promising alternative to monoclonal antibodies.^24^ All these compounds induced a significant improvement of CD8 T cell functions, with a slightly better effect of the TLR8 agonist over the other compounds. The effect of the different modulatory agents was greater in low EI patients, namely those with predominance of less exhausted T cell phenotypes.

Although very promising, our results suffer, however, from a major flaw, since our analysis was based on a single epitope specificity. To overcome this drawback, first we tried to select and characterise also polymerase-specific CD8 T cells though they were detected only at very low frequencies and in a limited number of viraemic CHB patients. A simultaneous phenotypic profiling of pol_455-463_- and core_18-27_-specific CD8 T cells was performed in some CHB patients and, in line with previous studies,^14–16^ we observed higher levels of TOX and lower levels of PD-1 in pol_455-463_-specific CD8+T cells. These findings highlight intrinsic differences in exhaustion profiles between pol_455-463_ and core_18-27_-specific CD8 T cells in patients with chronic active hepatitis, making the EI score not applicable to pol_455-463_-specific CD8 T cells.

As a second strategy to overcome the single specificity issue, we then investigated whether the subset distribution detected among core-specific CD8 T cells can actually reflect the features of the overall HBV multispecific CD8 T cell population. To do this, we analysed the phenotypic profile of the total CD8 T cell population, assuming that expression of exhaustion markers in the overall CD8 T cell population should be mostly contributed by the multispecific repertoire of the different HBV antigenic specificities. Remarkably, the same stratification of chronic patients with different levels of functional T cell impairement, as defined by the calculation of the EI score for HBV core-specific CD8 T cells, was reproduced by staining total CD8 T cells with PD-1, CD127, TOX and Bcl-2. This means that a simple and easy to be performed phenotypic assessment of the overall CD8 T cell population may allow to predict individual patient’s responsiveness to in vitro modulation without the need of virus-specific T cell analysis.

In order to validate the predictive value of the total CD8 T cell phenotypic profile on the response to immune modulation, we then used a different cohort of patients and a different experimental approach looking at the effect of immune modulatory compounds on multispecific CD8 responses induced in vitro by PBMC stimulation with overlapping peptides covering the entire core and polymerase proteins. Results indicate that the phenotypic cut-off value derived from the initial series of experiments with core_18-27_-specific CD8 T cells can be applied to total CD8 T cells from a randomly selected cohort of CHB patients to identify infected hosts who are more or less likely to respond in vitro to immune modulatory compounds. Moreover, these experiments show that CD8 T cell functional recovery by immune modulation is effective also in the context of multispecific T cell responses stimulated by entire HBV proteins. Indeed, functional improvement was observed among both pol-specific and core-specific T cells with a major contribution of the latter to the overall recovery of HBV-specific T-cell responses.

In conclusion, our study demonstrates that co-expression profiling of specific exhaustion and memory markers by HBV core-specific and total CD8 T cells can allow to generate a predictive score suitable for the identification of distinct CHB patient cohorts with various degrees of CD8 T cell dysfunction and different susceptibility to functional T cell restoration in vitro by immune modulatory treatments. A crucial limitation, however, is that the predictive value of the EI score has been tested only in vitro and that clinical studies have not been performed so far to understand whether this simple phenotypic score can have an application in the clinical setting. This is particularly relevant because without a clinical assessment we cannot know whether the in vitro effect of the tested compounds can be reproduced in vivo and whether the level of restoration detected in vitro can actually translate into a clinically meaningful functional improvement. In favour of our prediction score based on a phenotypic profiling of total CD8 T cells is its simplicity of application which makes it a promising basis for the development of clinically useful T cell-based predictors. Indeed, a clinical trial with mitochondrial antioxidant compounds is being organised in CHB patients to characterise their effect in vivo and to validate the clinical value of our phenotypic CD8 T cell score.

## Data Availability

All data relevant to the study are included in the article or uploaded as online supplemental information.
