# Supplementary material for: Phenotypic CD8 T cell profiling in chronic hepatitis B to predict HBV-specific CD8 T cell susceptibility to functional restoration in vitro
Source: Gut. 2023 Jan 30;72(11):2123–37. doi: 10.1136/gutjnl-2022-327202 (PMC10579518; doi:10.1136/gutjnl-2022-327202)
Supplement: Supplementary data [file gutjnl-2022-327202supp001.pdf]

Phenotypic CD8 T cell profiling in chronic hepatitis B to predict HBV-specific CD8 T cell susceptibility to functional restoration in vitro

Marzia Rossi, Andrea Vecchi, Valeria Barili, Camilla Tiezzi, Paola Fisicaro, Amalia Penna, Ilaria Montali, Stephane Daffis, Simon P. Fletcher, Anuj Gaggar, Jonathan Medley, Michael Graupe, Latesh Lad, Alessandro Loglio, Roberta Soffredini, Marta Borghi, Teresa Pollicino, Cristina Musolino, Arianna Alfieri, Federica Brillo, Diletta Laccabue, Marco Massari, Chiara Boarini, Gianluca Abbati, Giuseppe Pedrazzi, Gabriele Missale, Pietro Lampertico, Carlo Ferrari, Carolina Boni.

Table of contents

Supplementary materials and methods.......... 2

Supplementary results.......... 8

Supplementary figures.......... 8

Table S1.......... 11

Rossi M, *et al. Gut* 2023; 72:2123–2137. doi: 10.1136/gutjnl-2022-327202

SUPPLEMENTARY MATERIALS AND METHODS

Study subjects

Patients were enrolled at the Units of Infectious Diseases of Parma, at the Unit of Internal Medicine of the Modena University Hospital and at the Fondazione IRCCS Ca' Granda Ospedale Maggiore Policlinico, Milan. The study was approved by the competent local Ethic Committees and all patients provided written, informed consent.

All patients were negative for anti-hepatitis C virus, hepatitis delta virus, human immunodeficiency virus type 1 and type 2 antibodies and for other markers of viral or autoimmune hepatitis.

A total of 376 chronic patients were assessed for HLA-A2 expression; 160 of them resulted HLA-A2+. $Core_{18-27}$-specific CD8 T cells were detectable in 60. Seventy-one HLA-A2+ patients were tested also with a polymerase-specific dextramer containing the pol sequence 455-463 and 23 of them tested positive. All patients who showed the presence of at least one of the two CD8 T cell epitopes tested (64 patients) were enrolled for subsequent analyses (table 1). They are divided as follows:

These 64 patients were divided as follows:

1. 35 treatment-naive CHB patients (total lack of virus control: HBsAg positive, anti-HBc positive, HBeAg negative/anti-HBe positive, elevated/fluctuating serum HBV-DNA levels and alanine aminotransferase values);
2. 16 immune subjects with spontaneous HBsAg clearance and anti-HBs seroconvertion (complete virus control: HBsAg negative, anti-HBs positive, anti-HBc positive, serum HBV-DNA negative) following a long lasting chronic carriage of the virus (either active or inactive);

3. 13 NUC resolved patients (complete virus control: HBsAg negative, anti-HBs positive, anti-HBc positive, serum HBV-DNA negative) with HBsAg clearance following long-term NUC therapy;
4. 7 healthy subjects as controls.

Additional 24 HLA-A2 negative treatment-naïve CHB patients were enrolled and studied to further validate the association between phenotype of total CD8 T cells and T cell responsiveness to modulatory compounds (table 2).

At the time of analysis, some individuals in the "CHB" group had low HBV-DNA and ALT levels because of the widely fluctuating profile of virus replication and liver inflammation.

Peptides and dextramers

To evaluate virus-specific CD8 T cells responses in HLA-A2 positive patients, peptides corresponding to the HLA-A2-restricted core 18–27 (FLPSDFFPSV) and polymerase 455–463 (GLSRYVARL) epitopes of genotype D HBV were used. As a control, a peptide containing the influenza A virus (FLU) matrix (GILGFVFTL) HLA-A2-restricted epitope was employed. Peptides were purchased from Proimmune (Oxford, UK). The PE-labeled dextramer peptide-HLA class I complexes corresponding to HBV-core and FLU matrix were purchased from Immudex (Copenhagen, Denmark). To study the T cell responses to variant HBV core 18-27 epitopes, mutated peptides and the corresponding HLA class I dextramers were synthesized according to sequencing data of HBV core 18-27 epitopes derived from each patient (see Table 1).

PBMC were also stimulated with a panel of 15-mer peptides, overlapping by 10 residues, covering the HBV core and polymerase genotype D sequences, pooled in 3 mixtures (core, pol 1, pol 2), as previously described [18].

Rossi M, *et al. Gut* 2023; 72:2123–2137. doi: 10.1136/gutjnl-2022-327202

Phenotypic analysis of HBV-specific CD8 T cells

PBMCs were isolated from fresh heparinized blood by Ficoll-Hypaque density gradient centrifugation and cryopreserved in liquid nitrogen until the day of analysis.

After PBMCs staining with surface markers (CD3, CD8, PD-1, CD127, CD39), the Fixation/Permeabilization and Permeabilization Buffers were used according to the manufacturer's instructions with antibodies specific for TOX, TCF-1 and BCL-2. Details are provided in the supplementary table 1 of this manuscript. All determinations were performed using LRS Fortessa (Becton Dickinson, BD, Immunocytometry System, CA, USA). The data were processed with the FACS-DIVA or Flow-Jo software (BD, Becton Dickinson).

***Ex vivo* functional assessment of HBV-specific CD8 T cells.**

While viral peptides represent the most physiologically-relevant stimulus to study antigen-specific CD8 T cell responses, peripheral HBV-specific CD8 T cells in CHB patients typically show a very limited response to HBV peptides *ex vivo*. Therefore, we performed preliminary experiments to evaluate various stimuli acting at different levels of the T cell signaling cascade (anti-CD3/anti-CD28, PMA/ionomycin, IL2, IL12). Based on these studies (data not shown), we selected PMA/ionomycin stimulation for functional T cell characterization experiments.

After one hour PBMC stimulation with PMA (phorbol 12-myristate 13-acetate, 100ng/ml) and ionomycin (1µg/ml), brefeldin-A (BFA, 10 µg/ml, BD, Becton Dickinson) was added for the last 3 hours. Then, cells were washed and surface-stained with dextramer-PE and fluorochrome-conjugated antibodies (Supplementary table 1). PBMC were fixed, permeabilized and stained with cytokine antibodies according to the manufacturer's instructions. Samples were acquired on a BD LSR Fortessa and analyzed with the FlowJo

Rossi M, *et al. Gut* 2023; 72:2123–2137. doi: 10.1136/gutjnl-2022-327202

software. Data were expressed as the total frequency of IFN-γ, TNF-α single positive and IFN-γ/TNF-α double positive dextramer+ CD8 T cells.

***In vitro* T-cell expansion and treatment with immunomodulators.**

Short-term T-cell lines were generated by 8-10 days PBMCs stimulation either with the $core_{18}$-$_{27}$ peptide (1 μM) or with a panel of 187 15-mer peptides (1 μM), overlapping by 10 residues, covering the HBV core and polymerase genotype D sequence, pooled in 3 mixtures (core, pol 1, pol 2) alone or in the presence of the different tested compounds, including the polyphenolic compound Trans-Resveratrol (5 or 10 μM, Sigma-Aldrich, Missouri, USA), the mitochondria-targeted antioxidant MitoTempo (10 or 100 μM, Sigma-Aldrich), an anti-PD-L1 antibody (5 or 10 μg/ml, Invitrogen, Clone MIH1), a small PD-L1 inhibitor molecule (concentrations of 0.5-5 μM, PD-L1 SM, GS-418, kindly provided by Gilead Sciences, Inc., Foster City, CA) and a selective TLR8 agonist (concentrations of 0.5-10 μM, Selgantolimod, provided by Gilead Sciences, Inc., Foster City, CA). In experiments with anti-PD-L1, the small PD-L1 inhibitor molecule and the TLR8 agonist, PBMCs were pre-incubated for 45 minutes with each compound and then stimulated with the peptides (1 μM).
To study the effect of mutations on the HBV-specific CD8 T cell function, PBMCs were stimulated for 10 days in vitro with WT- or variant-specific core 18-27 peptides (1 μM).

Direct Sequencing Analysis.

Serum DNA extracts were amplified by PCR using oligonucleotide primers specific for HBV DNA sequences flanking the entire preC/C genomic region (HBV1F, 5'-AAGACTGGGAGGAGTTGGG - 3' and HBV2R, 5'-ACCTTATGAGTCCAAGGAATACTAACA-3') and the Expand High Fidelity PCR System (Roche Diagnostics) according to the manufacturer's instructions. Nucleotide sequences of

PCR products were determined using the BigDye Terminator Cycle Sequencing Ready Reaction kit (Thermo Fisher Scientific) according to the manufacturer's instructions. The sequencing products were resolved in an automatic DNA sequencer (ABI PRISM 3500 Dx Genetic Analyzer; Thermo Fisher Scientific).

Statistical methods.

The GraphPad Prism software Version 7.00 and JASP Version 0.9.2.0 were used for statistical analysis. Normality distribution of data was tested by the Kolmogorov-Smirnov test. Differences between multiple patient groups were evaluated by Kruskal-Wallis non-parametric test and the p-values were calculated and corrected for pair-wise multiple comparisons. Comparisons between two groups were assessed by the Mann-Whitney U test or the Wilcoxon-matched-paired test. Data correlations were evaluated by Spearman tests. The hierarchical-clustering analysis was performed by GeneSpring-GX software (Agilent Technologies) after a median baseline transformation of all samples. The Hierarchical Clustering Algorithm was calculated by normalized intensity values with Canberra Similarity Measure and Complete Linkage Rule. Up- and down-regulated parameters are represented in red and green, respectively.

HBV-specific CD8 T cells were stained with exhaustion (PD-1, TOX, CD39) and memory (CD127, Bcl-2, TCF-1) markers and analyzed by flow cytometry. Due to their own characteristics, expression values of the above markers can differ by orders of magnitude. Thus, comparisons or sums of these marker contributions are influenced by their different scale size and the largest contributions dominate over the other affecting the real importance of each variable. This problem can be overcome by normalization or standardization methods. A method widely used to express different variable scales on a common metric is the Z-score, a simple scale transformation that reduces an original set (population) of values with arbitrary

mean and arbitrary standard deviation to a set with zero mean and standard deviation equal to one. The transformation is accomplished by the formula z = (x-m)/s where “z” is the Z-score corresponding to the value x in the original set, “m” is the mean of the original set, and “s” is the standard deviation of the original set of data. The derived Z-values will exhibit mean equal to zero and standard deviation equal to one. If many variables are expressed as Z-score they can be compared on a common scale or can be used in the search of particular patterns in the data, such as, for instance, in cluster analysis or principal component analysis.

The suitable cut-off for the different CD8 T cell phenotypic profiles was obtained using a ROC curve analysis, where *Exhaustion Index* (low and high) was used as classification criterion. The optimal threshold points on the ROC curves were chosen as those maximizing the difference between true positive and false-positive rates, the so-called Youden’s index. The Youden index is the farthest point on the ROC curve from the line of equality (diagonal line). AUC (area under the curve) values were all close to 1, showing very high discriminating power. Calculations were performed with the statistical package IBM-SPSS v.26.

A dimensionality reduction algorithm (Flow-Jo software, BD), was applied to generate tSNE plot on flow cytometry values after data concatenation to generate a two-dimensional map of CD8 T cell samples.

Rossi M, *et al. Gut* 2023; 72:2123–2137. doi: 10.1136/gutjnl-2022-327202

SUPPLEMENTARY RESULTS

A higher frequency of the PD-1^{hi}CD127$^{low/-}$ T cell subset was visualized also by tSNE on multiparametric flow cytometry data in patients with high vs low EI (red area, figure S4). Moreover, the PD-1^{hi}CD127$^{low/-}$ T cell subset showed greater TOX, PD-1, CD39 but lower TCF1, CD127 and Bcl-2 staining intensity in patients with high compared to those with low EI (up-regulated expression level in red and down-regulated in blue; figure S4).

SUPPLEMENTARY FIGURES

Supplementary figure 1. Correlations of EI with ALT, viral load and HBsAg values in naive CHB patients. Statistics by the Spearman's correlation test.

Supplementary figure 2. Functional restoration of HBV-specific CD8 T cells by immune modulatory interventions. Percentage of IFNγ+ and TNFα+ CD8 T cells in short-term T-cell lines generated by core peptide stimulation in the presence or absence of Resveratrol (RSV), MitoTempo (MT), anti-PD-L1, a PD-L1 Small Molecule (PD-L1 SM) and a TLR8 agonist (TLR8a). Percentage of IFNγ+ and TNFα+ CD8 T cells in untreated and treated paired T cell samples from individual chronic patients is illustrated (CHB patients with high and low E.I., n=7 and n=13, respectively); statistics by the Wilcoxon-matched-paired test.

Supplementary figure 3. Correlations of EI with the responses to the distinct immune modulations. A) Correlation between EI values and percentage of double-positive IFNγ+TNFα+ CD8 T cells in short-term T-cell lines generated by core$_{18-27}$ peptide stimulation of PBMC from CHB patients in the presence of Resveratrol (RSV), MitoTempo (MT), anti-PD-L1, a small PD-L1 inhibitor molecule (PD-L1 SM) and a TLR8 agonist (TLR8a). B) Correlation between EI and Delta values of double-positive IFNγ+TNFα+ CD8 T cells

derived by subtracting CD8 T cell frequencies of untreated from treated short-term T-cell lines generated as in A). Statistics by the Spearman's correlation test.

Supplementary figure 4. Comparison of the immuno-stimulatory effects of the different modulatory compounds on HBV-specific T-cell responses. Percentage of IFNγ+, TNFα+ and double-positive IFNγ+TNFα+ CD8 T cells in short-term T-cell lines generated by core18-27 peptide stimulation in the presence or absence of Resveratrol (RSV), MitoTempo (MT), anti-PD-L1, a PD-L1 Small Molecule (PD-L1 SM) and a TLR8 agonist (TLR8a). Treatment-induced variations (difference between treated and untreated paired samples) of IFNγ+, TNFα+ and double-positive IFNγ+TNFα+ CD8 T cell frequencies are illustrated (black lines indicate the median percentage of cytokine producing CD8 T cells). Each dot represents individual CHB patients. Statistically significant differences by Kruskal-Wallis with Dunn's multiple comparisons test.

Supplementary figure 5. Expression intensity of different exhaustion and memory/differentiation markers. The expression intensity of TOX, PD-1, CD39, TCF1, CD127 and Bcl-2 in each CD8 T cell population is illustrated by tSNE plots (up-regulation in red, down-regulation in blue). Data derive from four representative CHB patients with high or low EI (two subjects per group, 150,000 CD8 T cells for each patient). The red areas in the first two grey tSNE plots illustrate the PD-1^{hi}/$CD127^{low/-}$ T cell subset among the total CD8 T cell population.

Supplementary figure 6. Correlation between phenotypic CD8 T cell profiles and efficiency of individual responses to immune modulations. Responses of short-term CD8 T-cell lines generated by $core_{18-27}$ peptide stimulation of PBMC from CHB patients in the presence of the indicated immune modulatory compounds (RSV, MT, anti-PD-L1, PD-L1 SM and TLR8a) are illustrated as percentage of double-positive IFNγ+TNFα+ CD8 T cells within

the indicated CD8 T cell subsets (PD1^{hi}TOX^{hi}, PD1^{hi}TOX^{hi}Bcl2^{-}, PD1^{hi}TOX^{hi}CD127^{-}, PD1^{hi}TOX^{hi}TCF1^{-}). Statistics by the Spearman's correlation test.

Supplementary figure 7. Correlations between phenotypic profiles of total CD8 T cells and ALT, viral load or HBsAg levels in naive CHB patients. Statistics by the Spearman's correlation test.

Supplementary figure 8. Functional recovery of core and polymerase-specific CD8 T cells. A) Delta values of IFNγ+, TNFα+ and double-positive IFNγ+TNFα+ CD8 T cells in short-term T-cell lines generated by PBMC stimulation with pools of overlapping 15-mer HBV core or polymerase peptides (left and right panels, respectively) from chronic patients with high (n=15, orange) and low (n=17, blue) percentages of PD-1^{hi}TOX^{hi} CD8 T cells (see Figure 7 legend for further details); statistics by the Mann-Whitney test. B-C) Correlation between percentage of PD-1^{hi}TOX^{hi} CD8 T cells and percentage of IFNγ+, TNFα+ and double-positive IFNγ+TNFα+ CD8 T cells in short-term T-cell lines, generated as described above, illustrated as cumulative data of all treatments (B) and as numbers of immune modulatory agents able to induce a positive response (C). Statistics by the Spearman's correlation test.

Rossi M, *et al. Gut* 2023; 72:2123–2137. doi: 10.1136/gutjnl-2022-327202

Supplementary Table 1. Antibodies and Reagents

Name	Supplier	Cat no.	Clone no.
anti-CD3-BV 510	BD Biosciences, NJ, USA	564713	HIT3a
CD8-BV786	BD Biosciences, NJ, USA	563823	RPA-T8
CD127-PE-CF594	BD Biosciences, NJ, USA	562397	HIL-7R-M21
CD39-APC-Vio770	Miltenyi Biotec, Germany	130-105-532	MZ18-23C8
PD-1-PeCy7	Biolegend	329918	EH12.2H7
TCF1-Alexa488	Cell Signaling Technology	6444S	C63D9
Tox-APC	Miltenyi Biotec, Germany	130-118-335	REA473
Bcl-2-BV450	BD Biosciences, NJ, USA	560637	Bcl-2/100
CD8-APC-H7	BD Biosciences	560179	SK1
CD3-PerCp	Miltenyi Biotec, Germany	130-113-131	BW264/56
TNF-α-APC	Biolegend	502912	MAb11
IFN-γ-APC-R700	BD Biosciences, NJ, USA	564981	B27
anti-CD3-BD Horizon™ PE-CF594	BD Biosciences, NJ, USA	562280	UCHT1

Name	Supplier	Cat no.
Lymphocyte separation medium	Biowest, Nuaillé, France	L0560-500
Brefeldin A	ENZO Life Sciences	BML-G405-0025
RPMI 1640	EuroClone, Italy	ECM2001L
Brillant Stain Buffer	BD Biosciences, NJ, USA	563794
Trans-Resveratrol	Sigma, Missouri, USA	R5010-100MG
Selgantolimod (TLR8 agonist)	Kindly provided by Gilead Sciences, Inc., Foster City, CA	GS-256
Small molecule PD-L1 inhibitor	Kindly provided by Gilead Sciences, Inc., Foster City, CA	GS-418
MitoTempo	Sigma	SML0737-5MG
anti-PD-L1 antibody (clone MIH1)	Invitrogen	16-5983-82
Dimethyl sulfoxide	Sigma	D5879-L
10X D-PBS DULBECCO'S PHOSPHATE BUFFERED SALINE	EuroClone	ECM4004XL
HBSS	EuroClone	ECB4006L
FIX&PERM®Cell Fixation and Permeabilization Kit(CE)	NordicMUbio	GAS-002
Fetal Bovine Serum	CORNING, New York, USA	35-079-CV
TF Perm/Wash Buffer 5X	BD Biosciences, NJ, USA	51-90081 02
eBioscience™Fixation/Permeabilization Diluent	eBioscience	00-5223-56
eBioscience™Fixation/Permeabilization Concentrate	eBioscience	00-5123-43
IL-12	Sigma	I2276
IL-7	ThermoFisher, Massachusetts, USA	PHC0076
IL-2	ThermoFisher	PHC0021
Phorbol 12-myristate 13-acetate (PMA)	Sigma	16561-29-8

Rossi M, *et al. Gut* 2023; 72:2123–2137. doi: 10.1136/gutjnl-2022-327202
