# Supplementary figures and images for: Phenotypic CD8 T cell profiling in chronic hepatitis B to predict HBV-specific CD8 T cell susceptibility to functional restoration in vitro
Source: Gut. 2023 Jan 30;72(11):2123–37. doi: 10.1136/gutjnl-2022-327202 (PMC10579518; doi:10.1136/gutjnl-2022-327202)

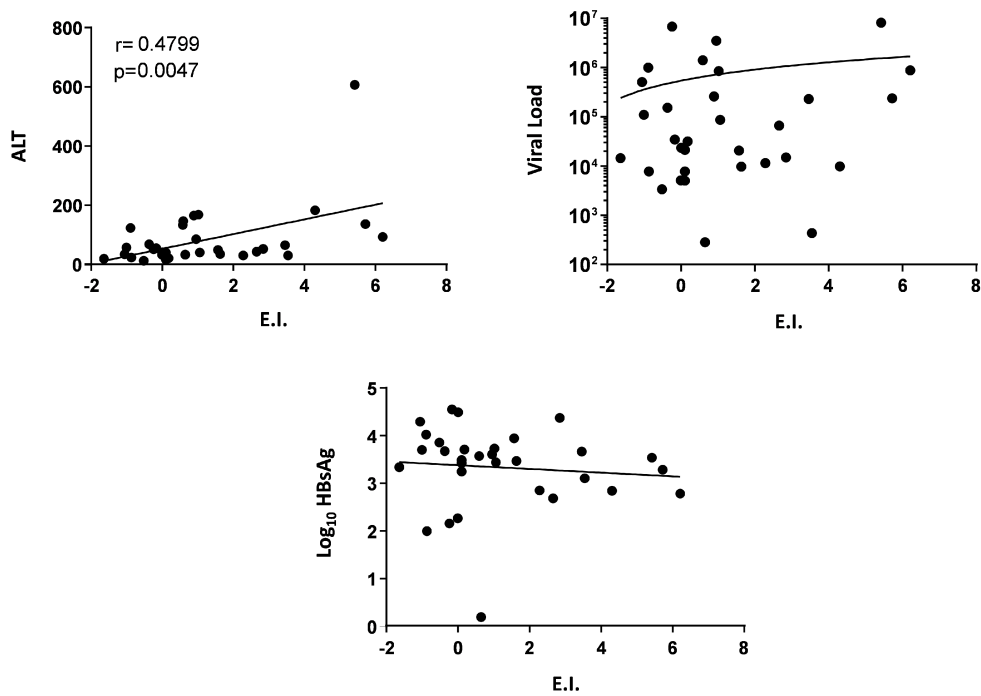

Supplement: Supplementary data [file gutjnl-2022-327202supp002.pdf]

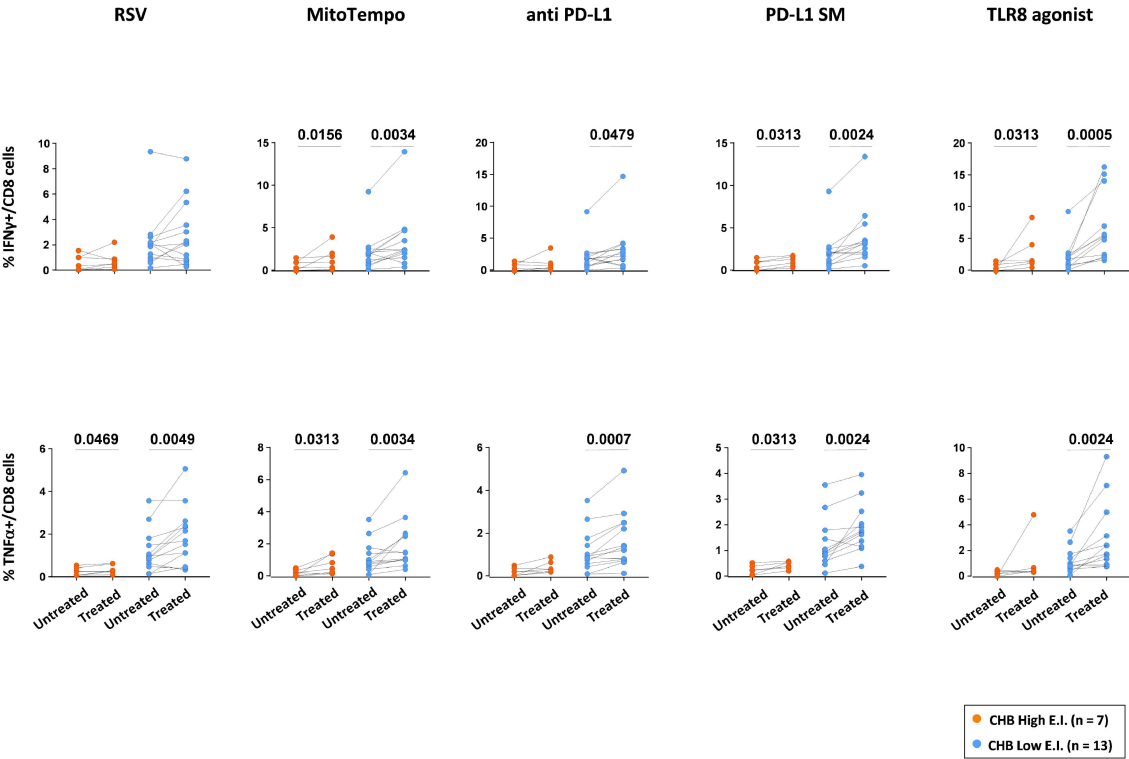

Supplement: Supplementary data [file gutjnl-2022-327202supp003.pdf]

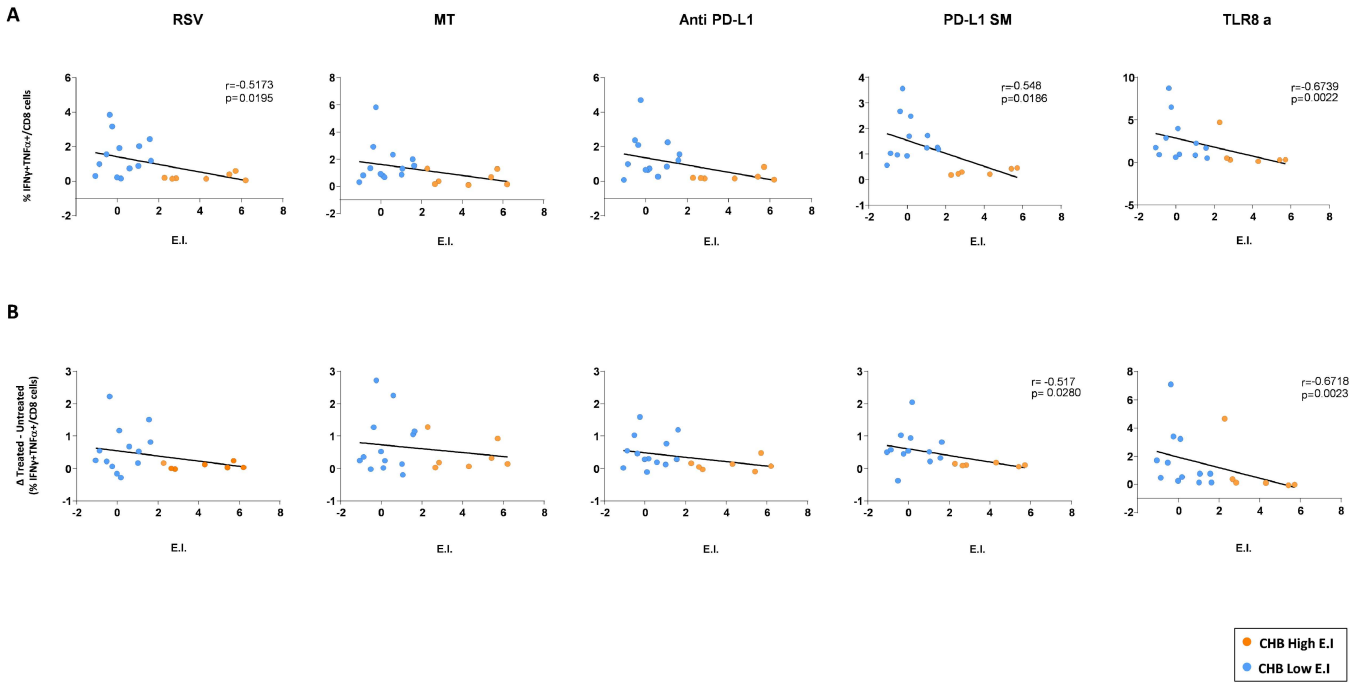

Supplement: Supplementary data [file gutjnl-2022-327202supp004.pdf]

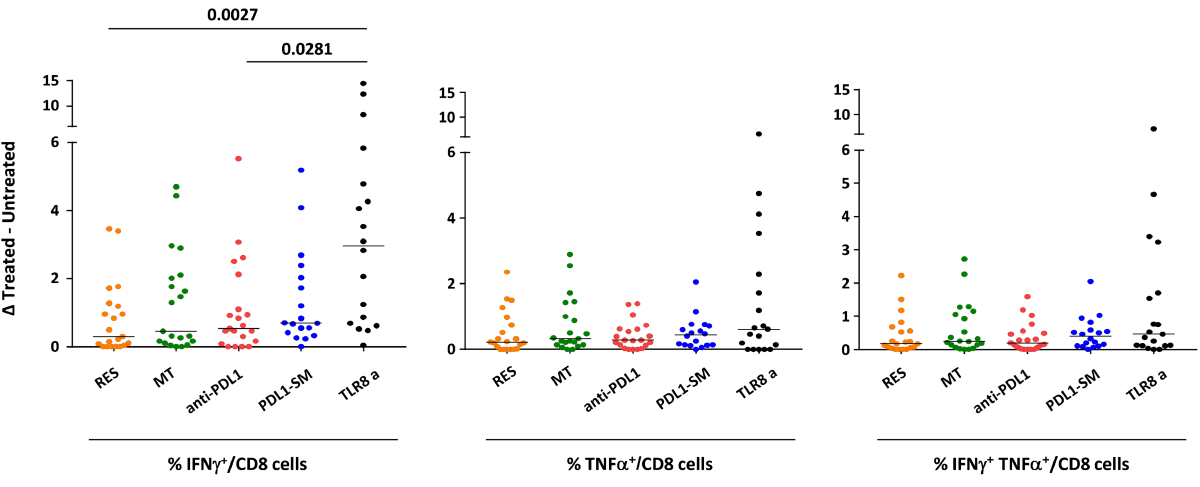

Supplement: Supplementary data [file gutjnl-2022-327202supp005.pdf]

■ Total CD8 + T cells  
■ PD1<sup>hi</sup> CD127<sup>low/-</sup>

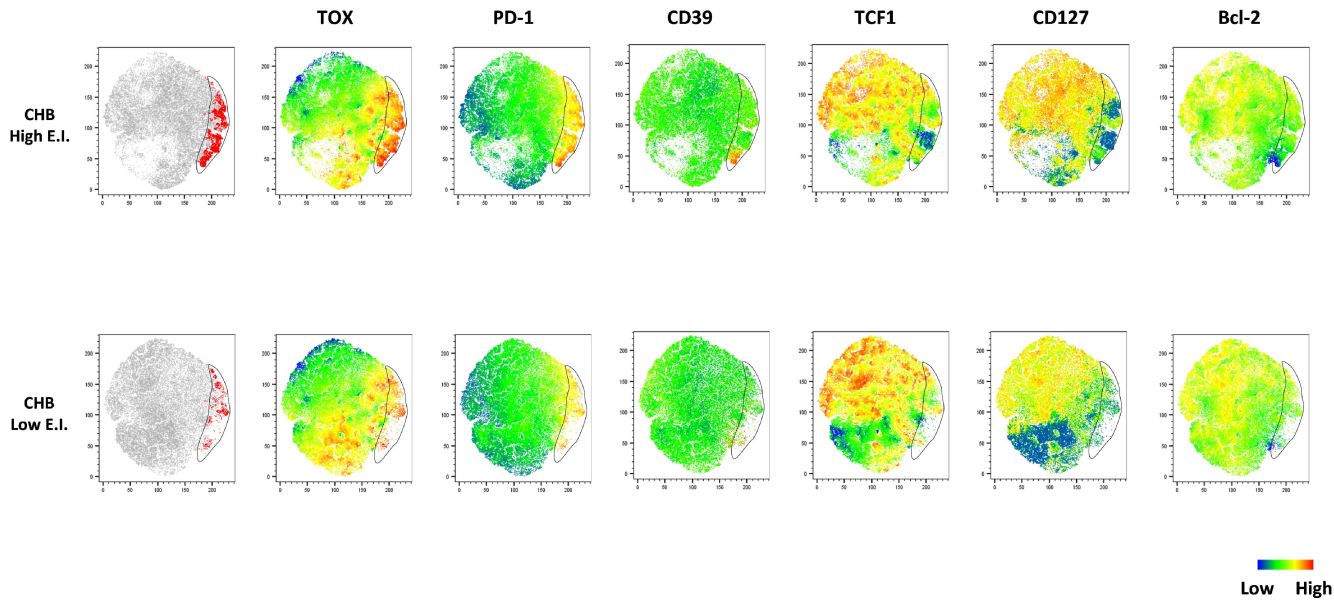

Supplement: Supplementary data [file gutjnl-2022-327202supp006.pdf]

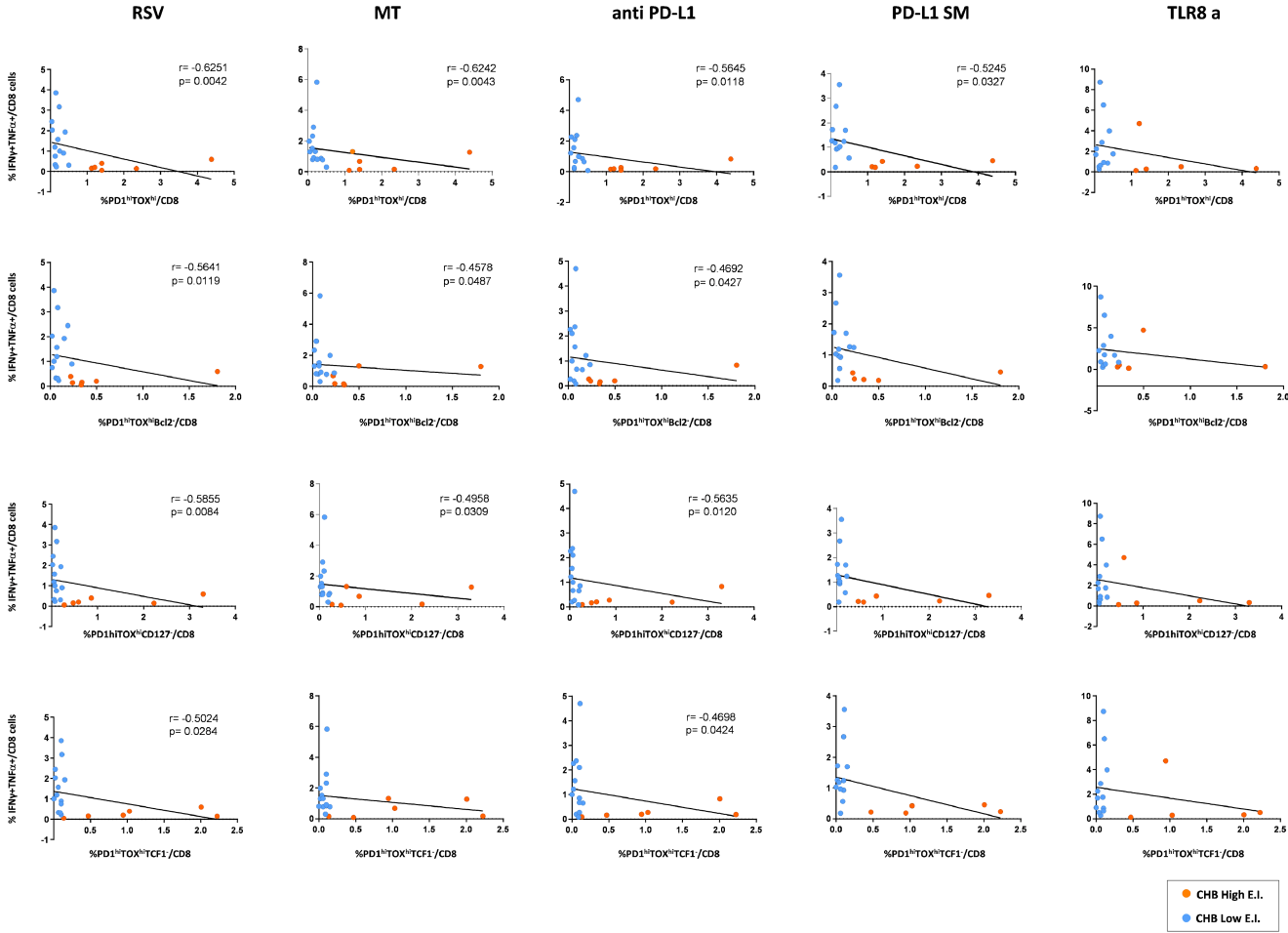

Supplement: Supplementary data [file gutjnl-2022-327202supp007.pdf]

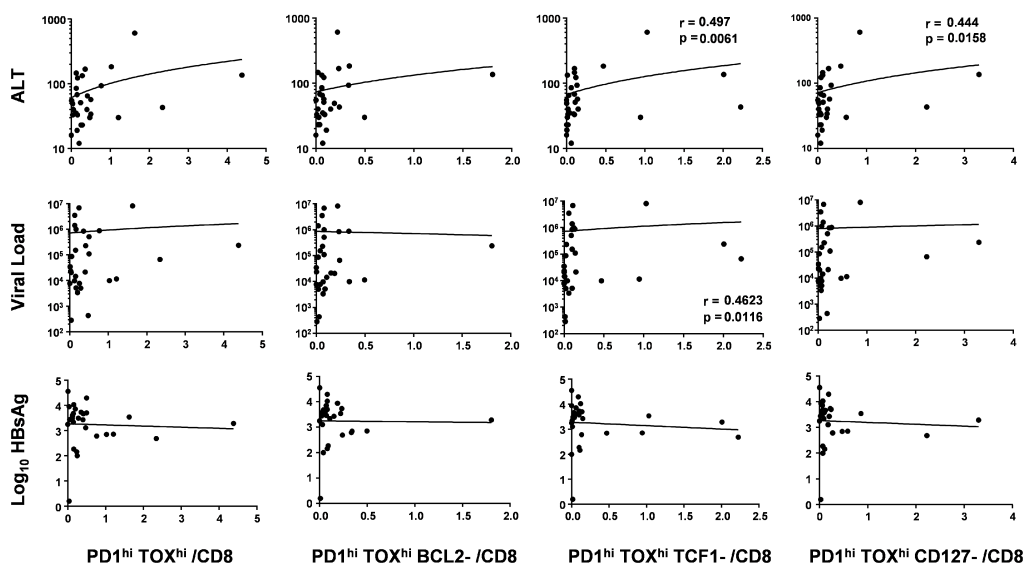

Supplement: Supplementary data [file gutjnl-2022-327202supp008.pdf]

CORE

POLYMERASE

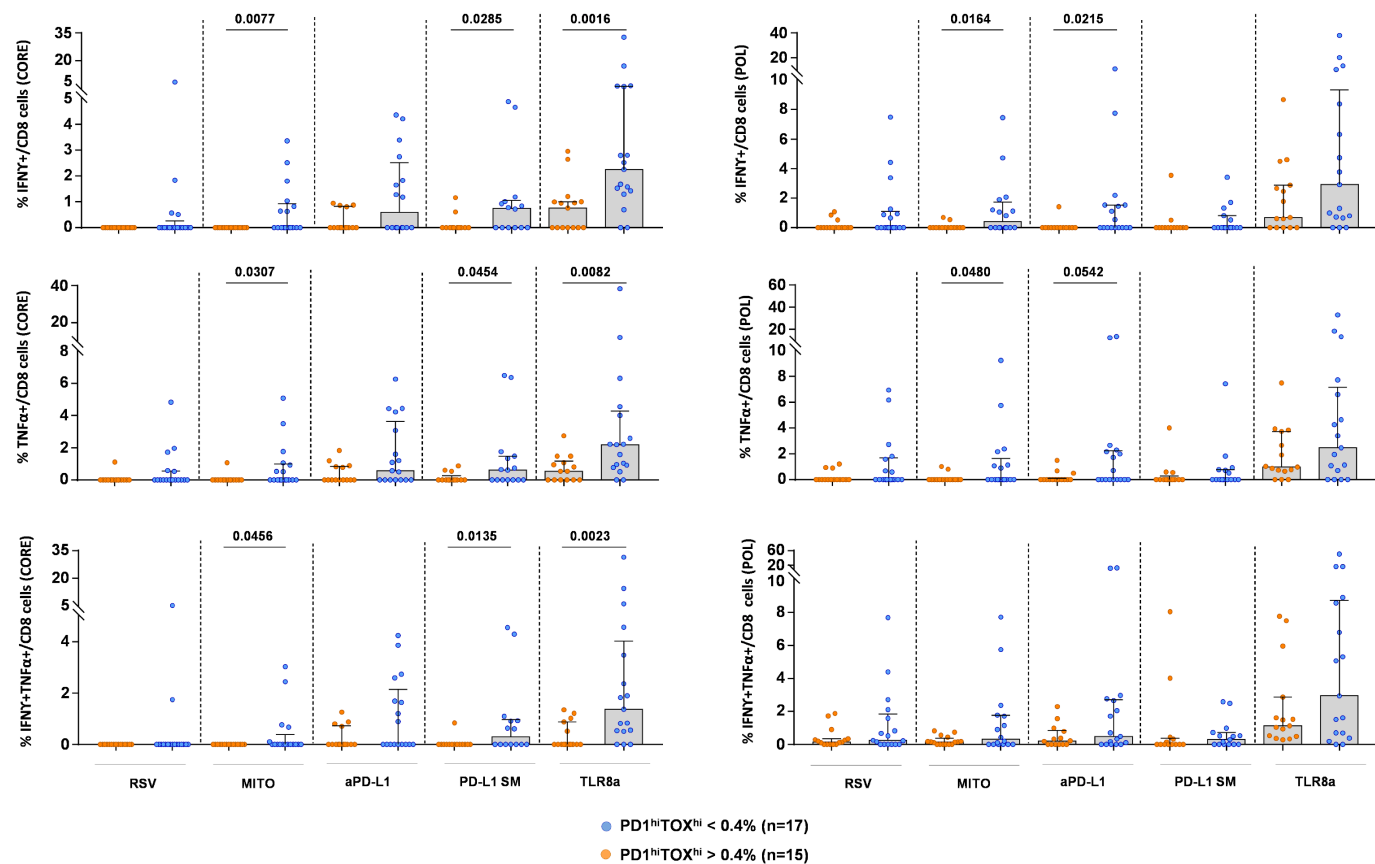

B

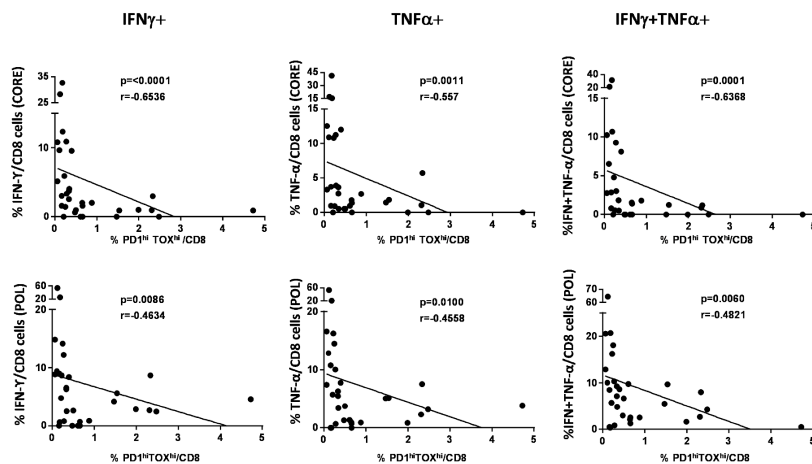

C

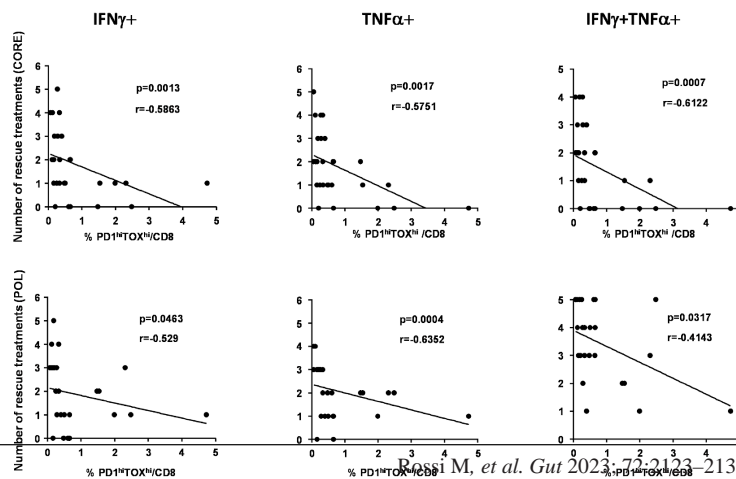

Supplement: Supplementary data [file gutjnl-2022-327202supp009.pdf]
